# Potential prognosis index for m^6^A-related mRNA in cholangiocarcinoma

**DOI:** 10.1186/s12885-022-09665-3

**Published:** 2022-06-07

**Authors:** Huaqiang Zhu, Haini Zhao, Jianlu Wang, Shuchao Zhao, Chaoqun Ma, Dongliang Wang, Hengjun Gao, Faji Yang, Qingqiang Ni, Hongguang Li, Xu Zhou, Chunqing Zhang, Jun Lu

**Affiliations:** 1 Department of Hepatobiliary Surgery, Shandong Provincial Hospital, Cheeloo College of Medicine, Shandong University, Jinan, 250021 Shandong China; 2 Department of Hepatobiliary Surgery, Shandong Provincial Hospital Affiliated to Shandong First Medical University, Jinan, 250021 Shandong China; 3 Jinan Health Publicity and Education Center, Jinan, 250021 Shandong China; 4 ChosenMed Technology (Beijing) Co., Ltd., Beijing, 100176 China; 5 Department of Gastroenterology, Shandong Provincial Hospital, Cheeloo College of Medicine, Shandong University, Jinan, 250021 Shandong China

**Keywords:** Cholangiocarcinoma, N6-methyladenosine, m^6^A-related mRNA, Risk model, Prognosis

## Abstract

**Background:**

Cholangiocarcinoma (CHOL) is a malignant tumor that originates in the extrahepatic bile duct and can extend from the hilar region to the lower end of the common bile duct. The prognosis of CHOL patients is particularly poor; therefore, in this study, we screened mRNAs correlated with N6-methyladenosine (m^6^A) to construct a risk model for prognosis in CHOL.

**Methods:**

The TCGA-CHOL dataset was applied to obtain and analyze the coexpression of 1281 m^6^A-related mRNAs, from which 14 were selected for further analysis through univariate proportional hazards (cox) regression analysis. Aryl hydrocarbon receptor interacting protein (*AIP),* CCAAT/enhancer binding protein beta (*CEBPB*)*,* syndecan1 (*SDC1*)*,* vacuolar protein sorting 25 homolog (*VPS25*) *and* syntaxin binding protein 2 (*STXBP2*) were then screened out through the least absolute shrinkage and selection operator (LASSO) and multivariate Cox regression analysis to develop a precise m^6^A-related mRNA prognosis risk model (MRMRPM) with an area under curve (AUC) of 0.908 and 0.923 after 1 and 2 years, respectively. We divided the samples into high-risk and low-risk groups using the m^6^A-related mRNA prognosis risk model.

**Results:**

Kaplan–Meier analysis indicated poor overall survival (OS) for the high-risk group. Two Gene Expression Omnibus (GEO) datasets (GSE89748 and GSE107943) were used to validate the risk model. The results of drug sensitivity and immune cell infiltration analysis showed that the risk model could serve as a prognosis index of potential immunotherapeutic characteristics and drug sensitivity. Furthermore, the proportion of resting dendritic cells and regulatory T cells was positively associated with an increased expression of four m^6^A-related mRNAs — *AIP, CEBPB, SDC1, and VPS25* — in the high-risk CHOL group.

**Conclusions:**

Our findings suggest that this model can be a prognostic indicator for CHOL patients.

**Supplementary Information:**

The online version contains supplementary material available at 10.1186/s12885-022-09665-3.

## Background

Cholangiocarcinoma (CHOL), which originates from epithelial cells in the bile duct, is anatomically divided into intrahepatic and extrahepatic cholangiocarcinoma [1]. The morbidity and mortality of CHOL continues to increase and accounts for 7–10% of all hepatobiliary malignancies [2]. The lack of specific early diagnostic markers and molecular targets for therapy means that most CHOL patients are diagnosed at an advanced stage and radical surgical resection can treat only 20% of patients [3]. These factors indicate that the 2-year OS is only 5.5% today [4]. The recent increase in research on cholangiocarcinoma has led to the discovery and elucidation of the expression characteristics and mechanisms of some key molecules involved in the development of cholangiocarcinoma, providing a new basis for targeted molecular therapies.

m^6^A is a widespread modification of the mRNA base that involves methylation of the sixth N position and generally affects adenosine (A). This modification maintains the stability of mRNA and is directly involved in the migration and proliferation of tumor cells [5–7]. The importance of this modification in gene regulation has generated considerable research interest in m^6^A.

Several studies have indicated that m^6^A genes regulate the formation and development of tumors. The high expression of methyltransferase-like proteins (*METTL*)*3* in hepatocellular carcinoma (HCC) induces degradation of the tumor suppressor gene suppressor of cytokine signaling 2 (*SOCS2*) via m^6^A modification and is associated with poor prognosis [8]. *METTL3* is also highly expressed in bladder cancer, and mediated target genes form a multilevel regulatory network that promotes tumor growth [9]. In recent years, several studies have suggested that the abnormal regulation of the m^6^A genes is related to CHOL [10, 11]. As the most common mRNA modification, transcriptome studies had linked m^6^A to cancer development, affecting the self-renewal and differentiation, proliferation, apoptosis, invasion and metastasis, resistance, and immune suppression processes of tumor stem cells [12–15]. Therefore, m^6^A-related mRNA is a potential molecular target for cancer treatment and drug development. However, the correlation between m^6^A-related mRNA, immune infiltration, and clinical prognosis in CHOL patients remains unclear.

In our study, the mRNA expression profiles, including expression data for 21 m^6^A genes, related to 36 CHOL samples were obtained from The Cancer Genome Atlas (TCGA) database. mRNAs related to the m^6^A genes were first screened based on Pearson’s correlation analysis. The m^6^A-related mRNA expression and prognostic information of the 36 TCGA-CHOL cohorts were then applied to establish an individual prognostic risk model for CHOL and the prognostic risk model was validated using two GEO datasets. We then analyzed the tumor mutational burden (TMB) of each TCGA-CHOL cohort. The sensitivity of 83 candidate drugs was explored using external cell lines from the Cancer Cell Line Encyclopedia (CCLE) to obtain drug targets for a risk model based on the relationship between the five obtained mRNAs and the m^6^A genes. We found that the relationship between the immunotherapy response and m^6^A-related mRNAs could promote infiltration by immune cells in CHOL. Thereafter, a model was constructed using the five m^6^A-related mRNAs to predict the OS of CHOL patients through nomogram analysis.

## Methods

### Database of CHOL patients

All data used to construct the m^6^A-related mRNA risk model were obtained from the TCGA database (https://portal.gdc.cancer.gov/). Testing sets were downloaded from the GEO database on the GEO website (http://www.ncbi.nlm.nih.gov/geo).

### Identification of m^6^A genes and m^6^A-related mRNAs

The expression database of 21 m^6^A genes and all protein-coding mRNAs were extracted from the TCGA database. m^6^A-related mRNAs were selected using the Pearson correlation coefficient method to calculate correlations between m^6^A and other genes (*R >* 0.3 and *p <* 0.001). A total of 1281 m6A-related mRNAs were screened.

### Analysis of function and protein-protein interaction

The functions of m^6^A-related mRNAs with different expression were determined by Gene Ontology (GO) and Kyoto Encyclopedia of Genes and Genomes (KEGG) pathway analyzes with *p*-values < 0.05 [16–18]. The online database STRING (https://string-db.org/) was used to construct the PPI network.

### Identification and validation of the MRMRPM model

The m^6^A-related mRNA model was developed utilizing 36 TCGA-CHOL samples for training and validated using the GSE89748 and GSE107943 datasets that were downloaded from GEO. We screened m^6^A-related mRNA from 1281 m^6^A-related mRNAs (*p <* 0.05) via univariate Cox regression [19] and used LASSO with 10-fold cross validation to find seven m^6^A-related mRNAs [20]. The five-m^6^A-related mRNA risk model was then established through multifactor cox regression. The formula MRMRPM = Ʃ (βi × EXPi) was used to calculate the risk score based on multivariate Cox analysis.

### Analysis of candidate target genes for transcription factors

The candidata target genes of each transcription factor were extracted according to cotarget genes from the ChEA ChIP-X target gene database (https://maayanlab.cloud/Harmonizome/dataset/CHEA+Transcription+Factor+Targets) [21].

### Principal component analysis

PCA was used to perform model recognition, dimensionality reduction, and visualization of the groups associated with the whole gene expression, m^6^A gene expression, and the expression of five m^6^A-related mRNA to further verify the discriminative ability of the model [22].

### Kaplan–Meier survival analysis

Kaplan–Meier analysis was performed using the “survival” package. We did not use any value to exclude the survival time, because the sample was too small.

### Drug sensitivity prediction targeting for the m^6^A-related mRNA model in clinical treatment

The CCLE gene expression data were used to predict the drug sensitivity for MRMRPM and the sensitivity of patients to 83 drugs embedded in the pRRophetic package was predicted by the gene expression in the CHOL samples. The difference in the prognostic groups predicted by the model in terms of their sensitivity to drugs was then analyzed, combined with the two risk groups.

### Investigation of the m^6^A-related mRNA model regarding immunotherapeutic treatment

First, we counted the TMB in each TCGA-CHOL sample to produce the total amount of coding variants/the length of exons (38 million), and we then divided the 36 samples into high- and low-TMB groups. Second, we evaluated the abundance of immune cells in the 36 TCGA-CHOL samples using CIBERSORT (https://cibersort.stanford.edu) with *p <* 0.05. We then analyzed the correlation between m^6^A-related mRNA expression and the infiltration by immune cells.

### Establishing and validating the predictive nomogram

Comparing other clinical characteristics (clinical stage, age, sex, and risk value), we validated MRMRPM by multivariate and univariate Cox regression analyses of the TCGA-CHOL patients.

Furthermore, a nomogram for MRMRPM was established using 1- and 2-year OS, combined with other clinical indicators (clinical stage, age, sex, and risk value).

### Statistical analysis

The R software clusterProfiler package and the survival ROC R software package were used to perform gene functional enrichment analyses and the ROC curve, respectively. The Student’s *t*-test was used to assess significant differences.

## Results

### Profiles of m^6^A-related mRNAs in CHOL

**Fig. 1:**
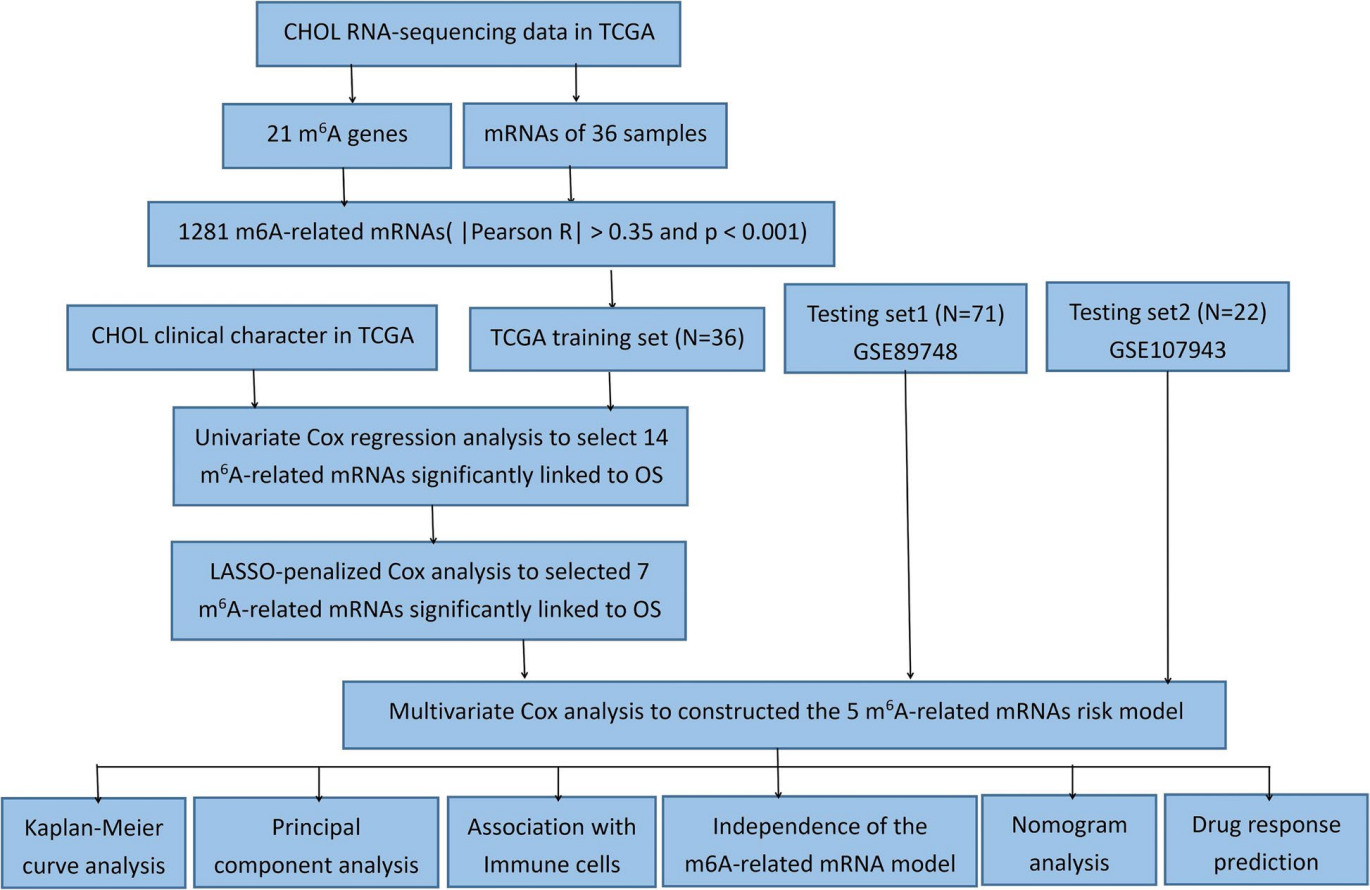
Workflow chart for this study

**Fig. 2:**
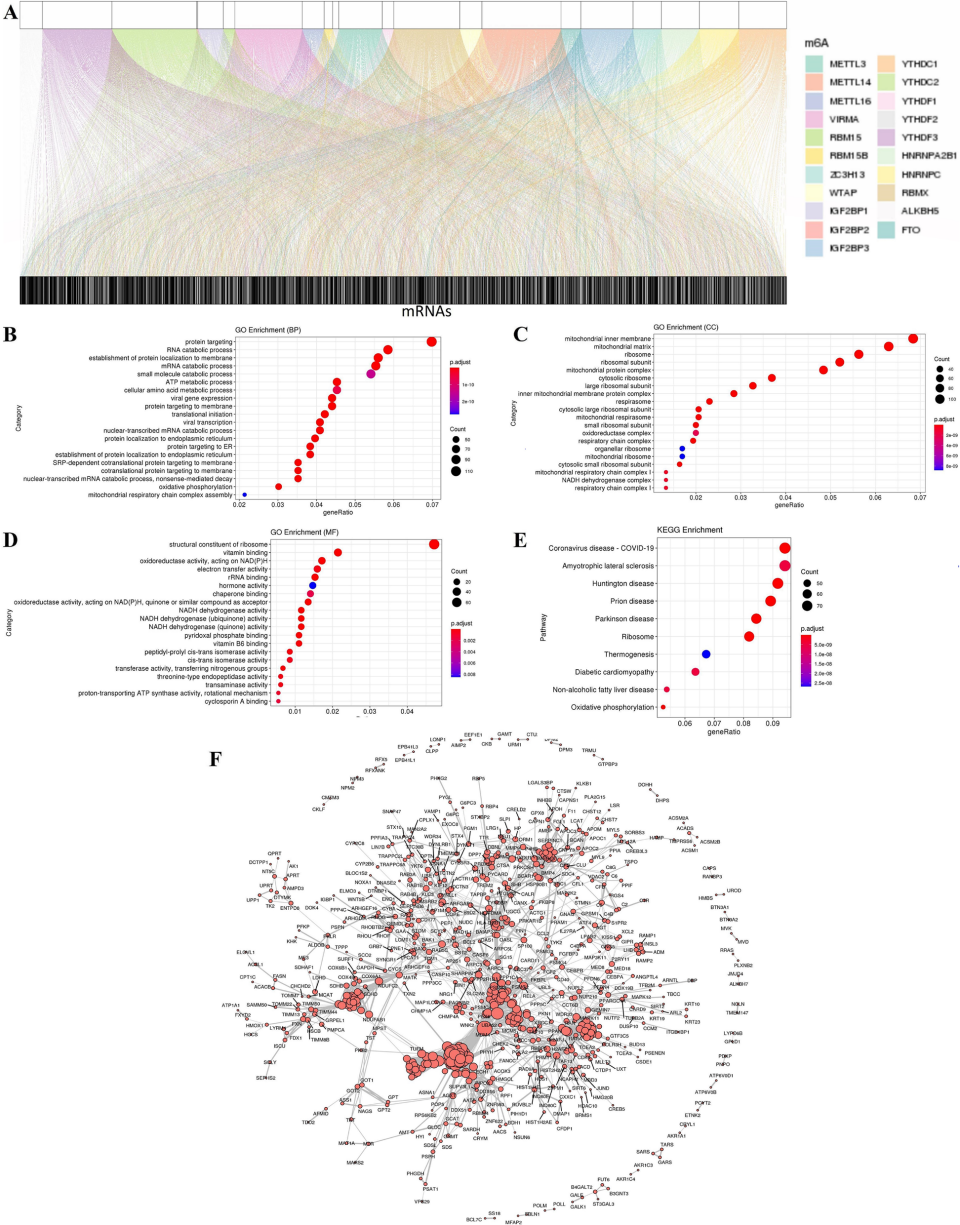
Identification of m^6^A-related mRNAs in Cholangiocarcinoma patients and enrichment pathway analysis**. A** Sankey relational diagram of 21 m^6^A genes and m^6^A-related mRNAs from 36 samples in TCGA, and |Pearson R| > 0.35 and *p <* 0.001 analysis with the m^6^A-related mRNA significance filtering threshold. **B-D** Function analysis of GO enrichment in differentially expressed m^6^A-related mRNAs. **E** Immune-related pathways involved in the differential expression of m^6^A-related mRNAs by KEGG pathway analysis. **F** PPI network of 57 proteins. TCGA: The Cancer Genome Atlas; GO: Gene Ontology; KEGG: Kyoto Encyclopedia of Genes and Genomes; PPI: protein-protein interaction

The research process is presented in Fig. 1. The profiles of 21 m^6^A gene expressions and the mRNA expression in 36 samples were downloaded from the TCGA-CHOL dataset (Table 1). Data from 16 (44%) males and 20 (56%) females were included in the study. There are no differences between male and female in the sample, and indicated that there is no specific bias in gender and tumor stage for our study (Table 1). m^6^A-related mRNAs associated with at least one of the 21 m^6^A genes were defined using the Pearson correlation coefficient (*R >* 0.35 and *p <* 0.001). The coexpression network obtained for m^6^A-mRNA is presented in Fig. 2A, and the 1281 identified m^6^A-related mRNAs are presented in Supplementary Table S1. GO analysis indicated that in terms of biological processes (Fig. 2B), RNA catabolic process, protein targeting, mRNA catabolic processes, the establishment of proteins on membranes, small molecule catabolic processes, ATP metabolic processes, and cellular amino acid metabolic processes were enriched in samples, whereas m^6^A-related mRNAs were found mainly in the mitochondrial protein complex, the inner mitochondrial inner membrane, inner mitochondrial membrane protein complex, mitochondrial matrix, ribosomal subunit, cytosolic ribosome, large ribosomal subunits, and mitochondrial ribosomes within cells (Fig. 2C). In terms of molecular function, m^6^A-related mRNAs were mainly involved in producing the structural constituents of ribosomes (Fig. 2D). Following GO analysis, we analyzed data from the Kyoto Encyclopedia of Genes and Genomes (KEGG) to show that m^6^A-related mRNA was also implicated in COVID-19 and amyotrophic lateral sclerosis. Therefore, the datasets obtained indicate that m^6^A-related mRNAs were mainly related to biological functions and metabolic pathways of ribosomes and mitochondria (Fig. 2E). We then analyzed PPI networks using the STRING database to produce the interaction network in Fig. 2F. Isolated points, namely m6A-related mRNAs without interactions, were removed from the network. The maximum connected subgraph in the network was then extracted using the *igraph* [23] package to obtain 57 hub genes.

**Table 1:** Summary of patient characteristics

	Alive (*n =* 18)	Dead (*n =* 18)	Total (*n =* 36)	*P*-value
Gender				
Male	8 (44%)	9 (50%)	16 (44%)	0.9959
Female	10 (56%)	9 (50%)	20 (56%)	
Age				
Mean (SD)	63 (13.4)	63 (12.7)	63 (12.8)	
Median [Min, Max]	68.5 [29, 82]	65 [31, 81]	66.5 [29, 82]	
Stages I–II	15 (83%)	13 (72%)	28 (78%)	0.69
Stages III–IV	3 (17%)	5 (28%)	8 (22%)

### Construction prognostic model according to m^6^A-related mRNAs in CHOL

**Fig. 3:**
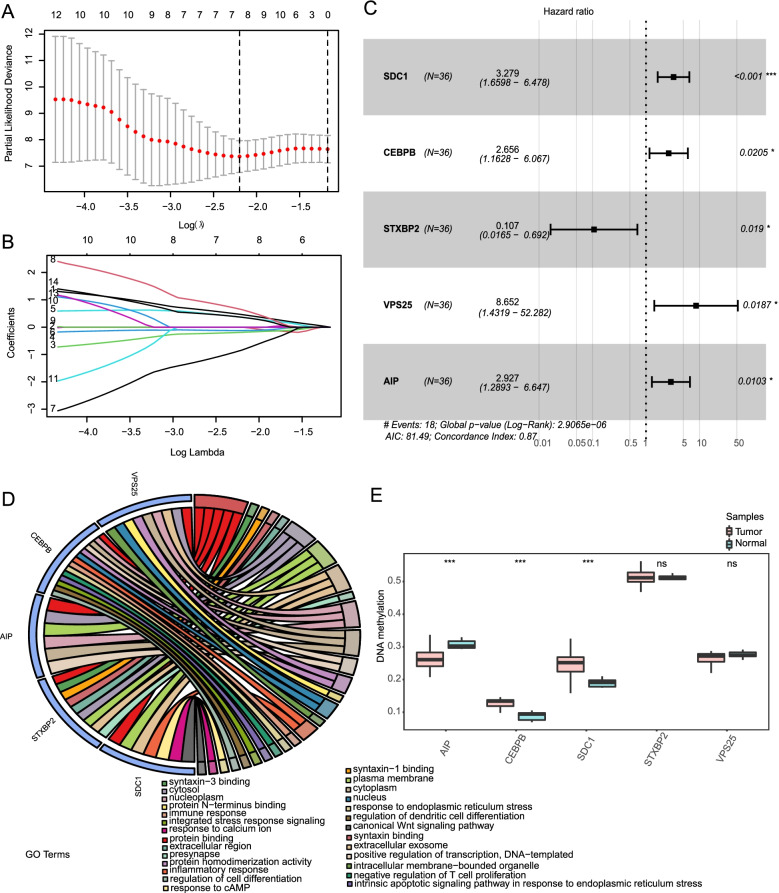
Risk model for patients with Cholangiocarcinoma based on m^6^A-related mRNAs. **A** LASSO regression coefficient and lambda correspondence diagram, with LASSO coefficient profiles of the characteristics against the log (λ). **B** LASSO regression, partial-likelihood deviance curve with log (λ). The figure above shows lambdas. Min (collimated dashed line on the left) and lambdas.1se (vertical dashed line on the right). The value above represents the number of features included in the model with the associated λ value. **C**) The patient risk ratio of the five m^6^A-related mRNAs is shown in the forest map. **D**) GO enrichment analysis of the five m^6^A-related mRNAs. **E** methylation analysis of the five m^6^A-related mRNAs. LASSO: least absolute shrinkage and selection operator; GO: Gene Ontology

Univariate Cox regression analysis of m^6^A-related mRNAs was performed with the R package “*survival*.” Them^6^A-related mRNAs were considered associated with a prognosis and therefore applicable for subsequent analysis when *p <* 0.05. Fourteen m^6^A-related mRNA genes from the 1281 m^6^A-related mRNAs associated with CHOL were significantly related to OS (Table 2). LASSO was used to select a variable optimization model and LASSO Cox analysis was conducted using the significant m^6^A-related mRNAs to construct a risk prognosis model [20]. The 10-fold cross-validation method was used to eliminate collinearity in gene optimization and simplified models. The variable λ in LASSO was utilized to find the best model. As λ increased, the regression coefficient β for each variable decreased, and a λ of 0 indicates that a variable contributes little to the model and could be eliminated. The LASSO screening indicated that seven m^6^A-related mRNAs were suitable for constructing the risk prognosis model and were used in subsequent studies (Fig. 3A, B). LASSO analysis was followed by multivariate Cox analysis and five characteristic m^6^A-related mRNA prognosis biomarkers for CHOL patients were obtained (Fig. 3C, Table 3). The relative hazard ratio (HR) for the five m^6^A-related mRNAs are presented in a forest plot (Fig. 3C). The MRMRPM was constructed based on the expression of the five m^6^A-related mRNAs, from which the risk to each patient was obtained. The expression of the five m^6^A-related mRNAs was statistically related to clinical characteristics; neither *SDC1* nor the *CEBPB* showed any such correlation. Subsequently, we found that *AIP* and *SDC1* were target genes of the transcription factor *CEBPB* from the ChEA ChIP-X target gene database (Supplementary Table S2). We also analysed the function of the five genes (Fig. 3D, E), The result of GO enrichment showed that all of the five mRNAs were involved in protein binding (Fig. 3D), and methylation analysis indecated that normoal tissue has higher methylation level of *AIP*, the methylation level of *CEPBP* and *SDC1* was increased in the normoal tissue, and there was no difference of methylation level of *VPS25* and *STXEBP2* (Fig. 3E).

**Table 2:** Univariate Cox analysis reveals 14 m^6^A-related mRNA results significantly associated with prognosis

Variable	Beta	HR	HRlower	HRupper	p-value
*SDC1*	0.68	1.97	1.16	3.32	0.01
*ELAC1*	−1.38	0.25	0.09	0.73	0.01
*CAMK1D*	−0.54	0.58	0.38	0.90	0.01
*OLFML3*	−0.56	0.57	0.35	0.93	0.02
*CEBPB*	0.65	1.91	1.07	3.39	0.03
*NARF*	1.34	3.83	1.12	13.15	0.03
*STXBP2*	−1.60	0.20	0.05	0.88	0.03
*VPS25*	1.43	4.17	1.11	15.60	0.03
*UBC*	−1.07	0.34	0.12	0.94	0.04
*PPP1CA*	1.26	3.53	1.06	11.75	0.04
*MRPL11*	0.86	2.36	1.03	5.41	0.04
*APOBEC3F*	−0.71	0.49	0.24	0.99	0.05
*DBNL*	−1.60	0.20	0.04	0.98	0.05
*AIP*	0.70	2.02	1.01	4.05	0.05

Note: Variable: gene, Beta: coefficient, HR: risk ratio, P: significance

**Table 3:** Five m^6^A-related mRNAs used to construct the risk prognosis model

Variable	coef	exp (coef)	SE (coef)	z	Pr(>|z|)	Significance
*SDC1*	1.19	3.28	0.35	3.42	0.00	***
*CEBPB*	0.98	2.66	0.42	2.32	0.02	*
*STXBP2*	−2.24	0.11	0.95	−2.35	0.02	*
*VPS25*	2.16	8.65	0.92	2.35	0.02	*
*AIP*	1.07	2.93	0.42	2.57	0.01	*

Notes: coeff, coefficient; ***p < 0.001; *p < 0.05

**Fig. 4:**
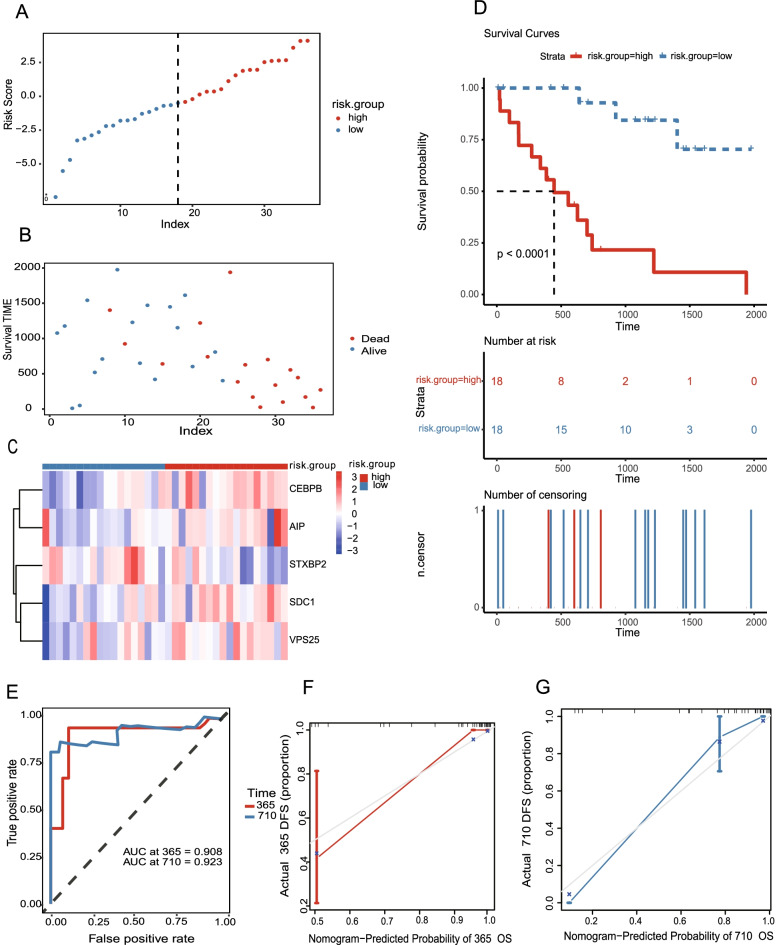
Estimation, evaluation, and calibration in training set for the five m^6^A-related mRNAs risk prognostic model **A**, **B** Distribution of the risk score for the five m^6^A-related mRNAs in the risk model. **B** Dot plot of the different survival time patterns for each risk group. **C** The different expression of m^6^ A-related mRNAs in each patient are shown in the heatmap. **D** Kaplan–Meier analysis of CHOL patients indicated that patients in the low-risk group had better OS. **E** The 1-year (AUC = 0.928) and 2-year (AUC = 0.903) ROC curves show the superior accuracy prediction of five independent prognoses in the m^6^A-related mRNA model. **F, G**) Calibration curves for survival after 1 y (**F**) and 2 y (**G** using MRMRPM. CHOL: Cholangiocarcinoma; OS: overall survival; AUC: area under curve; ROC: receiver operating characteristics; MRMRPM; five m^6^A-related mRNAs risk prognostic model

CHOL patients were then divided into high-risk (*n =* 18) and low-risk groups (*n =* 18) using the median risk as a threshold. The distribution of the low-risk and high-risk groups is presented in Fig. 4A, and the interrelation between the risk score and the survival period of CHOL patients in the two risk groups is reflected along with the survival status in Fig. 4B. The relative expression levels of the five m^6^A-related mRNAs for each patient in each risk group are shown in Fig. 4C. The expression of four of the five m^6^A-related mRNAs (excluding *STXBP2*) was increased in the high-risk CHOL patients (Fig. 4C). The patterns observed in the m^6^A-related mRNA expression were in accordance with the HR values (Table 3). Kaplan–Meier analysis indicated that high-risk CHOL patients had worse OS than low-risk CHOL patients (Fig. 4D). Supplementary Fig. S1 shows that high expression of the four m^6^A mRNAs (except *STXBP2*) (Supplementary Fig. S1E) was associated with worse OS in CHOL patients. The curves for receiver operating characteristics (ROC) that predict 1 and 2-year OS indicate that the model has an accurate risk prediction, with AUCs of 0.908 and 0.923, respectively (Fig. 4E). The 1- and 2-year OS predictions were calibrated using nomogram analysis, and all calibrated curves were well fit (Fig. 4F, G).

### Testing the prognostic capability of the constructed model

**Fig. 5:**
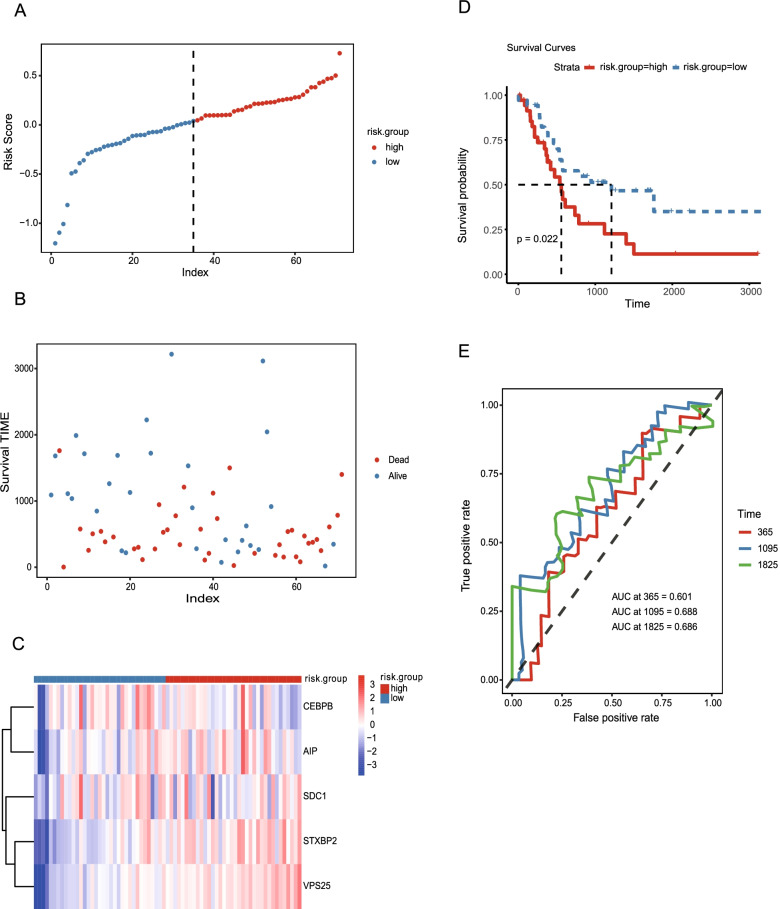
Validation of five m^6^A-related mRNAs risk prognostic model using the GEO testing dataset. **A**, **B** Distribution of the risk score of the m^6^A-related mRNA risk model in GSE89748. **B** Dot plot of different survival time patterns for each risk group in GSE89748. **C** The different m^6^ A-related mRNA expression in each patient in GSE89748 is shown in the heatmap. **D** Kaplan–Meier analysis of CHOL patients shows that low-risk groups in GSE89748 have better OS. **E** The 1-year (AUC = 0.601), 3-year (AUC = 0.688) and 5-year (AUC = 0.686) ROC curves show the accuracy in the prediction of the prognostic m^6^A-related mRNA model in GSE89748. GEO: Gene Expression Omnibus; GSE:gene set enrichment; CHOL: Cholangiocarcinoma; OS: overall survival; AUC: area under curve; ROC: receiver operating characteristics

**Fig. 6:**
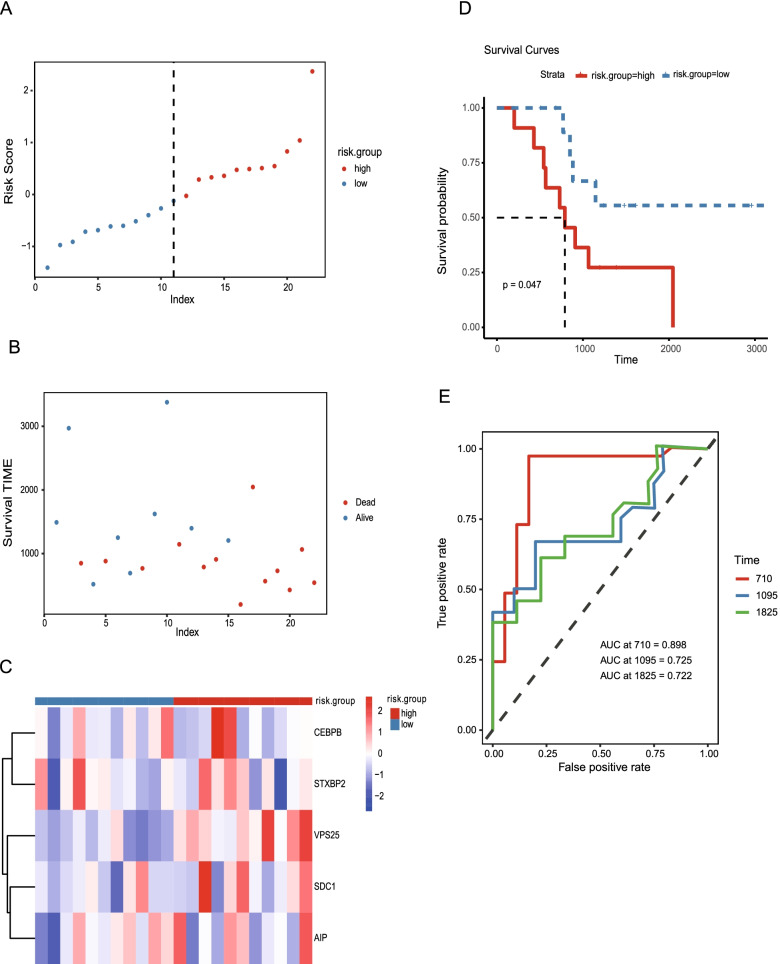
Validation of MRMRPM using the GEO testing dataset (GSE107943, *n =* 22). **A**, **B** Distribution of the risk score of five m^6^A-related mRNA risk models in GSE107943. **B** Dot plot of different survival time patterns for each risk group in GSE107943. **C** The different m^6^ A-related mRNA expression in each patient in GSE107943 is shown in the heatmap. **D** Kaplan–Meier analysis of CHOL patients shown that low-risk groups had better OS in GSE89748. **E** The 1-year (AUC = 0.898), 3-year (AUC = 0.725) and 5-year (AUC = 0.722) ROC curves show the accuracy in the prediction of the m^6^A-related mRNA model in GSE107943. MRMRPM; five m^6^A-related mRNAs risk prognostic model; GEO: Gene Expression Omnibus; GSE:gene set enrichment; CHOL: Cholangiocarcinoma; OS: overall survival; AUC: area under curve; ROC: receiver operating characteristics

Two GEO datasets; GSE89748 with 71 patients and GSE107943 with 22 patients, were used to validate the prognostic model in this study. The risk value for each CHOL patient in each dataset was calculated according to the uniform formula. CHOL patients in the GSE89748 (Fig. 5A-C) and GSE107943 datasets (Fig. 6A-C) were divided into high-risk and low-risk groups for the following analysis based on the expression of the five m^6^A-related mRNAs (Fig. 5A and 6A). The same expression pattern for the five m^6^A-related mRNAs was observed in the TCGA (Fig. 4C), GSE89748 (Fig. 5C), and GSE107943 (Fig. 6C) groups. Only *STXBP2* was upregulated in the low-risk group. Survival curves based on the two datasets also showed that the OS for CHOL patients was similar to that observed in the TCGA dataset and that the higher risk CHOL patients were associated with a worse OS than the lower risk CHOL patients (*p =* 0.022, Fig. 5D and *p =* 0.047, Fig. 6D). To validate the ability of the five m^6^A-related mRNAs risk prognostic model (MRMRPM), the conformance index and the area under the ROC curve (AUC) were also evaluated to achieve a risk score for the two datasets. The ROC curve for 1-, 3-, and 5-year OS prediction in the GSE89748 dataset suggest that the model possesses predictive accuracy with AUC values of 0.601, 0.688 and 0.681, respectively (Fig. 5E). Furthermore, the results of the analysis of the OS ROC curve at 1, 3, and 5 years indicated that the MRMRPM could accurately predict the prognosis of CHOL patients (AUC = 0.898, 0.725 and 0.722, respectively, Fig. 6E). As predicted, the OS and the ROC curves differed between the two risk groups, suggesting that the level of risk predicted by the MRMRPM accurately described the prognosis of CHOL.

### Verification of the five m^6^A-related mRNA risk model by principal component analysis (PCA)

**Fig. 7:**
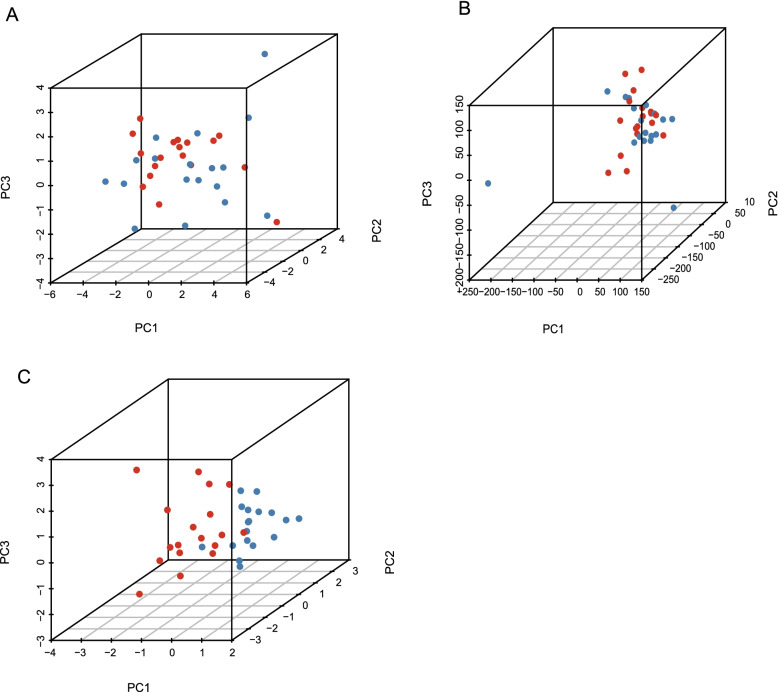
PCA between high-risk and low-risk groups. **A** 21 m^6^A genes. **B** entire gene expression profiles. **C**) 5 m^6^A-related mRNAs. PCA:principal component analysis

The entire gene expression profile, 21 m^6^A genes, the expression data for the five m^6^A-related mRNAs, the risk prognosis model generated using the m^6^A-related mRNA expression data, and the discrimination ability of the model were further verified by PCA (Fig. 7A–C). PCA performed on the expression levels of the characteristic mRNA (Fig. 7C) showed that the two risk groups differed, whereas the high-risk and low-risk individuals were randomly distributed over the two GEO datasets. These results verify the effective differentiating capacity of the characteristic five m^6^A-related mRNAs in the prognostic risk model.

### Testing drug susceptibility in the two risk groups

After the model was constructed and the differentiation ability of the model was verified by PCA, drug sensitivity was predicted to explore the difference between the prognosis in each group of patients as predicted by both the prognostic model and the drug sensitivity of the individuals involved.

**Fig. 8:**
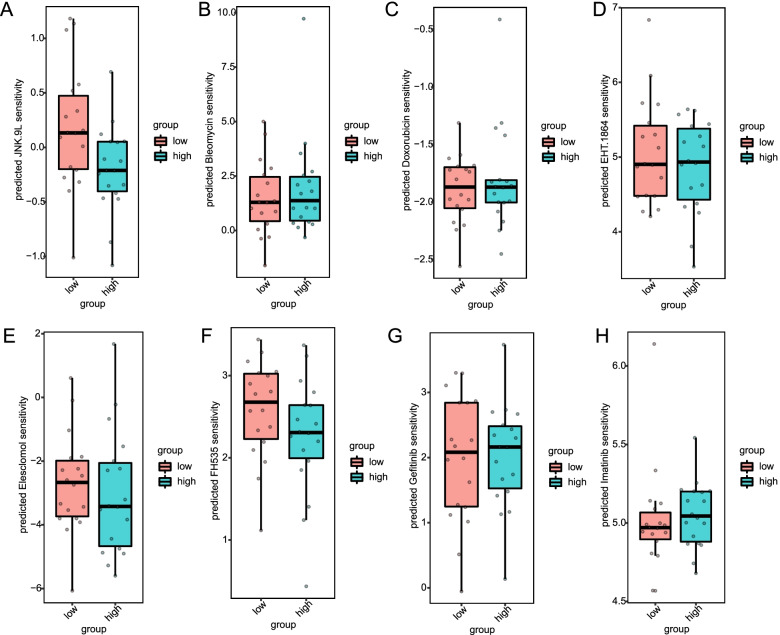
Drug sensitivity analysis for the m^6^A-related mRNA model. **A**-**H** Diagram of different sensitivities in the high- and low-risk groups for JNK.9 L (**A**), Bleomycin (**B**), Doxorubicin (**C**), EHT.1864 (**D**), Elesclomol **(E**), FH535 **(F**), Gefitinib **(G**) and Imatinib (**H**)

Gene expression data detailing the drug sensitivity of external cell lines (CCLE) [24] were used to predict drug susceptibility by estimating the sensitivity of patients to 83 drugs embedded in pRRophetic by genetic expression in CHOL samples (Supplementary Table S3). The difference between the groups of patients predicted by the prognostic model and their sensitivity to drugs was then analyzed with the high-low-risk groups. The low-risk TCGA-CHOL cohorts showed more sensitivity to the 83 drug compounds. Supplementary Table S3 and Fig. 8A-H show the results for c-Jun N-terminal kinase, Bleomycin, Doxorubicin, EHT.1864, Elesclomol, FH535, Gefitinib, Imatinib. The results suggest that 9 L (LNK. 9 L) might be used for patients with CHOL (*p <* 0.05, Supplementary Table S3 and Fig. 8A).

### TMB analysis of the two risk groups

**Fig. 9:**
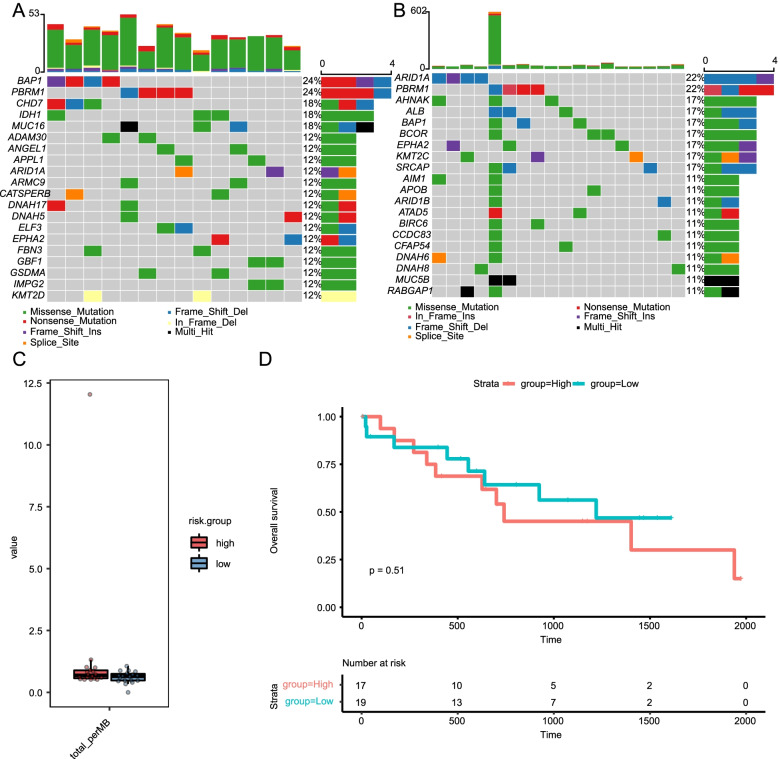
Statistics describing mutation and TMB in TCGA-CHOL samples. **A** Mutation information for the low-risk group is presented in the waterfall plot displays. **B** Waterfall plot displays mutation information for the high-risk group. **C** TMB differences observed in high- and low-risk patients. **D** Kaplan–Meier survival of TMB in CHOL patients. TMB: tumor mutational burden; TCGA: The Cancer Genome Atlas; CHOL: Cholangiocarcinoma

The somatic mutations of 36 CHOL patients were downloaded from the TCGA database and the mutations in the two risk groups analyzed (Fig. 9). Missense mutations were most frequently observed in both risk groups (Fig. 9A, B). The genes *BAP1* (24%), *PBRM1* (24%), *CHD7* (18%), *IDH* (18%), *MUC16* (18%), *ADAM30* (12%), *ANGEL* (12%), *APPL1* (12%), *ARID1A* (12%), and *ARMC9* (12%) (Fig. 9A) had the highest number of mutations in the low-risk group, whereas *ARID1A* (22%), *PBRM1* (22%), *AHNAK* (17%), *ALB* (17%), *BAP1* (17%), *BCOR* (17%), *BPHA2* (17%), *KMT2C* (17%), *SRCAP* (17%), and *SRCAP* (11%) had the highest number of mutations in the high-risk group (Fig. 9B). Meanwhile mutation in one of *ARID1A* and *PBRM1* were found in almost half of 32 intrahepatic cholangiocarcinomas through exomic sequencing [25]. *ARID1A* and *PBRM1* are involved in the formation of the BAF complex, and DNA damage in cells lacking *PBRM1* leads to cellular dynamic chromatin instability [26, 27]. Whether in high- and low- risk, the frequent alterations in *ARID1A* and *PBRM1* in cholangiocarcinomas highlight the key role of chromatin remodeling which in cholangiocarcinomas.

The TMB per million bases was then calculated for each of the 36 TCGA-CHOL patients; however, no significant differences were observed between the two groups in terms of this factor (Fig. 9C, Table 4) and no correlation was observed between the m^6^A-based classification index and the TMB. The survival curves suggest that the TMB of CHOL patients is not associated with OS (*p =* 0.51) (Fig. 9D).

**Table 4:** Results of the t-test and Wilcoxon analysis of the tumor mutation burden between the high-and low-risk groups in TCGA-CHOL cohort

Test method	*P* value	Adjusted P value	p.format	Significance
t test	0.25	0.25	0.25	Not significant
Wilcoxon	0.15	0.15	0.15	Not significant

To validate the sensitivity of MRMRPM to the immunotherapy response in CHOL, we next analyzed the relationship between the tumor immune microenvironment (IME) and the m^6^A-related mRNA model.

### Sensitivity of the m^6^A-related mRNA risk model to the immunotherapy response

**Fig. 10:**
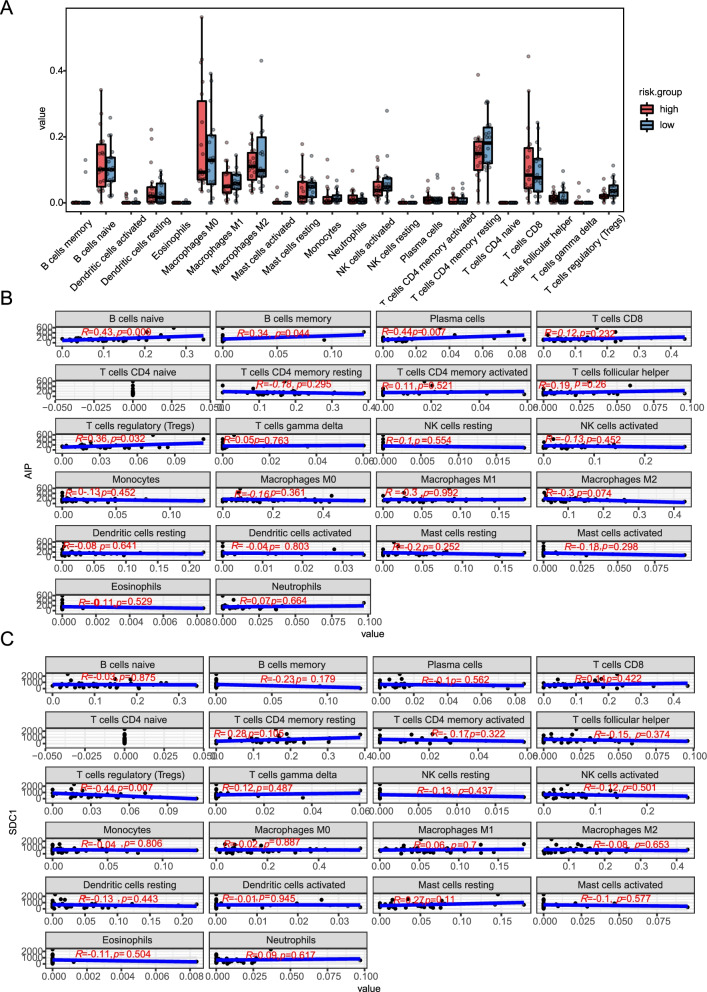
Relationship between abundance of TICs and five m^6^A-related mRNA expression in the two risk groups. **A** Violin plot shows the proportion of TICs in the high- and low-risk groups for CHOL. **B** Scatter plot shows the correlation between the 22 types of TICs and *AIP* expression. **C** Scatter plot shows the correlation between 22 types of TICs and *SDC1* expression. TICs: tumor-infiltrating immune cells; CHOL: Cholangiocarcinoma; *AIP*: aryl hydrocarbon receptor interacting protein; *SDC1*: syndecan1

To further confirm the correlation between m^6^A-related mRNA expression and tumor IME, CIBERSORT software [28] was used to analyze the infiltration profiles of 22 types of tumor-infiltrating immune cells (TICs) in the high-risk and low-risk groups. The fractions of the 22 immune cells were obtained by filtering with the “CIBERSORT” package, with *P >* 0.05 for each of the 36 TCGA-CHOL patients (Fig. 10A).

Moreover, our bioinformation analysis verified that the low-risk group had higher T cell regulatory (Tregs) infiltration levels (*p =* 0.023) than the high-risk group (Fig. 10A, Supplementary Table S4) and 22 kinds of TICs were associated with the expression of the five m^6^A-related mRNAs. Of the TICs, four types were positively associated with *AIP* expression, which is associated with memory B-cells, naïve B-cells, plasma cells, and Tregs. No TICs were negatively associated with *AIP* expression (Fig. 10B), whereas Tregs was negatively correlated with *SDC1* expression (Fig. 10C). Three kinds of TICs; monocytes, dendritic cells (DCs), and eosinophils, were positively associated with *CEBPB* expression. B-cell memory and naïve B-cells positively correlated with *AIP* expression showed a negative association with *VPS25* expression. Immune cell infiltration was not found to be related to the expression of the *STXBP2* gene. Only one incidence of correlation (positive or negative) was observed between the expression of 5 m^6^A-related mRNAs and immune cell infiltration in this study.

### Estimation of the m^6^A-related mRNA risk prognosis model for CHOL

**Fig. 11:**
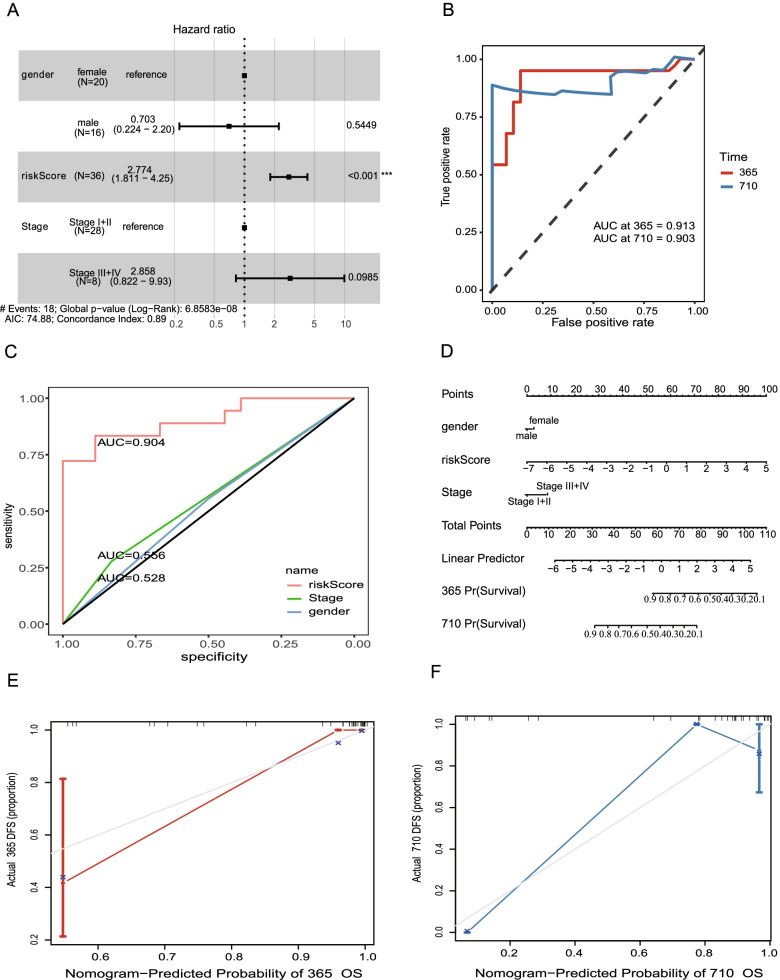
Estimate of MRMRPM and clinical characteristics in CHOL. **A** Hazard ratio of each independent risk signature is shown in the forest plot. **B**, **C** ROC curves of clinical characteristics and risk score to estimate prognostic sensitivity in CHOL. **D** Probability of 1- and 2-year OS by nomogram analysis. **E**, **F**) Calibration curves for 1-year (**E**) and 2-year (**F**) OS by MRMRPM. MRMRPM; five m^6^A-related mRNAs risk prognostic model; CHOL: Cholangiocarcinoma; ROC: receiver operating characteristics; OS: overall survival

The capability of independent prognostic signatures of the MRMRPM for CHOL was then estimated using univariate and multivariate Cox analyses. In this study, the higher risk score, HR, was based on univariate Cox analysis (HR = 2.72, 95% confidence interval (CI) = 1.77–4.18, *p <* 0.001) (Table 5) and multivariate Cox analysis (HR = 2.77, 95% CI = 1.81–4.25, *p <* 0.001) (Table 5, Fig. 11A), and the results obtained using the MRMRPM were not associated with tumor stage, gender, or age. The ROC curves for 1- and 2-year OS revealed that the MRMRPM could accurately predict the OS for the two CHOL groups with different risks (AUC = 0.913 and 0.903) (Fig. 11B). The AUC for risk was higher than other clinicopathological characteristics, and the risk was the only HR characteristic. The MRMRPM is therefore more reliable for use with CHOL (Fig. 11C). Compared to the other clinical signatures, the risk score obtained using the MRMRPM demonstrated significant predictive ability with nomogram analysis (Fig. 11D) and the calibrated curves suggest that the 1- and 2-year OS correction curves were consistent (Fig. 11E, F).

**Table 5:** Hazard ratio analyses of the clinical characteristics and risk score with the OS

Method	Term	Beta	HR	HRlower	HRupper	*p*-value
univariate Cox regression analysis	Risk Score	1.00	2.72	1.77	4.18	0.00
	Stage III + IV	0.39	1.48	0.52	4.21	0.47
	Gendermale	0.33	1.39	0.54	3.53	0.49
	age_at_index	0.01	1.01	0.97	1.05	0.62
multivariate Cox regression analysis	Gendermale	−0.35	0.70	0.22	2.2	0.54
	Risk Score	1.02	2.77	1.81	4.25	0.00
	Stage III + IV	1.05	2.86	0.82	9.93	0.10

## Discussion

Most patients with cholangiocarcinoma are diagnosed at an advanced stage. Over the past 25 years, the incidence and mortality of cholangiocarcinoma has increased, the prognosis of patients with cholangiocarcinoma has not improved significantly, and treatment is limited. The 5-year OS of cholangiocarcinoma is only 5–10%, and the median survival time of an individual with advanced cholangiocarcinoma is less than 12 months [2]. Therefore, it is vitally important that prognostic factors for CHOL are found. The Joint American Council on Cancer (AJCC) staging manual has become the benchmark for classifying cancers, predicting the prognosis, and determining the best treatment for cancer patients. However, the acceleration of cancer research has indicated that the AJCC-TNM stage system is not sufficient to estimate the prognosis or respond to the heterogeneity of these tumors. Even for patients with tumors at the same stage, the treatment response is highly heterogeneous, and other factors such as age, presentation status, and tumor site affect patient survival, which means that the information provided by the TNM staging system is limited in terms of prognosis [29]. Therefore, there is an urgent need to establish reliable prognostic biomarkers so prognoses can be improved. Recent studies investigating the use of screening for clinical prognostic mRNA indicators and other biomarkers have predicted the OS and the response of CHOL [30–32] and other cancers [33–36] to immunotherapy.

Higher TMB patients had better OS [35] when treated with ICIs. TMB is accompanied by an increase of tumor immune antigen, which is positively associated with an increasing CD8+ T cell count, and CD8+ T infiltration is the basis of immunotherapy. Previous studies have confirmed that an increase in the number of CD8+ T cells is not accompanied by an increase in the number of tumor antigens [37] for a variety of cancers, including breast and prostate. Analysis of data obtained from TCGA, the largest cancer gene database, indicates that not all cancers are accompanied by high CD8+ T-cell counts [38]. Thus, TMB-H tumor CD8+ T cells do not necessarily increase in response to the presence of tumors. McGrail et al. divided tumors into two categories based on the correlation with neoantigens; I: CD8 is positively correlated with neoantigens, and II: CD8 is not positively correlated with neoantigens. The results showed a significantly stronger immune checkpoint inhibitor response for high tumor mutation load (TMB-H) compared to low tumor mutation load (TMB-L) in category I cancers, whereas such effects were difficult to observe in category II cancers. In renal clear cell carcinoma and metastatic lung squamous cell carcinoma, TMB-L was found to lead to a prognosis better than TMB-H treated with immunotherapy [38]. After combining the data describing category II cancer, the objective response rate was only 15.3%, whereas the odds ratio of the objective response rate for TMB-L cancer was 0.46. TMB was significantly associated with the objective response rate in category I tumors, but not in category II tumors. The same result was observed in terms of OS, with a significant association detected for category I cancers and none for category II cancers. CHOL is a category II cancer [37], indicating that TMB is not prominently correlated with the objective response rate to immunotherapy in patients with this cancer. Marabelle et al. also found that TMB did not predict the efficacy of immunotherapy for CHOL patients [39]. Our study also revealed that TMB is not associated with OS (Fig. 9D), and no differences were observed in the OS of the high and low- TMB groups. These results indicate that TMB is not the only prognostic indicator of immunotherapy for CHOL patients and that other indicators should be considered in full. Therefore, we attempted to detect m^6^A-related mRNAs that contribute to survival and the classification of TNM stages in CHOL patients using the TCGA database.

The abnormal methylation of m^6^A RNA is closely related to the occurrence and development of tumors [40–42]. Although the molecular mechanism and role of m^6^A mediation in different tumors have not been fully elucidated, improvements in high-throughput sequencing and bioinformatics have led to the development of multiple methods for detecting and analyzing m^6^A methylation. The study of m^6^A and the associated participants that induce the process of reversible regulation (m^6^A-modifying enzymes and m6a-binding proteins) and the mechanisms involved in the occurrence and development of tumors have become a target for research [43, 44]. Data from the TCGA database showed that the mRNA expression of *METTL3* is significantly elevated in lung adenocarcinomas (LUAD) [45]. The mRNA metabolism includes mRNA processing, nuclear export, translation, degradation, and other processes and the mechanisms by which m^6^A is involved in mRNA metabolism are still being revealed. The mRNA processing promotes the maturation of precursor mRNA via three steps and studies have found that m^6^A is more abundant in the precursor than mature mRNA [46]. Many m^6^A writers, erasers, and readers are primarily located in cellular nuclear spots [47–49] and the selective splicing factors that play an important role in the splicing of precursor mRNA are also enriched in this subnuclear structure. Ribosomal development occurs in the cellular nucleoli, with cellular nucleolar proteins playing an important role in the transcription and processing of ribosomal RNA (rRNA). Dysfunctional ribosomal function usually leads to changes in the cell cycle and the occurrence of tumors. *METTL3* and *FTO* can influence the function of selective splicing factor 2 (*SRSF2*) by influencing the level of m^6^A, leading to regulation of the selective splicing process of mRNA [50]. In pancreatic adenocarcinoma research, a 16 m^6^A-related mRNA signature score system has been established for predicting the prognosis of pancreatic cancer patients based on the TCGA database [51]. In our study, we screened five m^6^A-related mRNAs from 1281 m^6^A-related mRNAs that utilized TCGA-CHOL cohorts for developing a MRMRPM. MRMRPM showed an accurate predictive ability with AUCs of 0.908 and 0.923 for the prediction of 1 and 2-year OS, respectively (Fig. 4E). Furthermore, we used two GEO datasets and visualization by PCA to validate the risk model and found that the MRMRPM model was accurate.

Among the five m^6^A-related mRNAs, *CEBPB* is involved in several of the most active biological processes associated with proliferation, such as biogenesis, the translation of ribosomes, and RNA processing [52]. The overexpression of *CEBPB* in C-REL-deficient myeloid-derived suppressor cell (MDSC) facilitates the expression of downstream *CEBPB* genes, enhancing mitochondrial respiration, weakening glycolysis, and inhibiting T cells and the immunotherapeutic response [53].

In this study, we found *AIP* and *SDC1* were downstream target genes of *CEBP* (Supplementary Table S2). *SDC1* is key in maintaining the survival and development of cancer cells [54], and *SDC1* expression has been related to tumor differentiation, depth of invasion, lymph node metastasis, distant metastasis, TNM stage, and prognosis [55, 56]. The AIP protein could induced Epstein-Barr virus [57] and the hepatitis B virus [58] by involveing in cell transformation. We found high-risk group had higher expression of *CEBPB, AIP* and *SDC1*. the overexpression of *CEBPB* might facilitated the overexpression of *SDC1* and *AIP* (Fig. 4C), resulted in worse OS in high-risk group in CHOL*.* Meanwhile, the expression of *CEBPB* and *STXEP2* was related TICs function [58]. Fto knockdown suppress the expression of transcription factors *c-Jun*, *JunB*, and *CEBPB,* restores the function of CD8+ T cells by impairing the glycolytic activity of tumor cells, thereby inhibiting tumor growth [59]. NK cells have severely reduced/absent degranulation and cytotoxicity in *STXBP2*-deficient patients [60]. In our study, the high-risk group had higher expression of *CEBPB* and lower *STXBP2* (Fig. 4C). The opposite pattern of expression might inhibited the activity of immune cells, thereby promoted tumor growth, resulted in worse OS in high-risk group in CHOL. More experiments are needed to confirm these conjecture.

Essential endocytoid sorting complex-II (ESCRT-II) is a preformed complex consisting of Vps36, Vps25 and Vps22 molecules and is recruited to endosomes [61, 62]. As an RNA-binding complex [63], ESCRT-II subcomplex can sort circRHOBTB3 into exosomes and secrete it out of the cell, resulting in tumor exosome escape mechanism [64]. In this study, the overexpression of *VPS25* in the high-risk group (Fig. 4C) promoted the formation of ESCRT-II and delayed or prevented the necrotic apoptosis of tumor cells, thus contributed to worse OS in high-risk groups (Fig. 4D). More experiments are needed to confirm this conjecture.

In the present study, CHOL patients with high expression of the four m^6^A-related mRNAs (except *STXBP2*) were associated with worse OS (Supplementary Fig. S1A-E), and CHOL patients with a higher risk score were also associated with worse OS (Fig. 4D). These results indicate that both the presence of m^6^A-related mRNA and the MRMRPM can be used as prognostic indices for CHOL patients.

We also compared the infiltration levels of different immune cells to further confirm the relationship between the expression of m^6^A genes, m^6^A-related mRNAs, and tumor IME. Early studies confirmed that higher expression of the m^6^A genes (*FTO*, *MELLT1*4, *METTTL3*, *YTHDC1*, *YTHDC2*, and *ZC3H13*) leads to higher immune infiltration, whereas patients with low immune infiltration showed higher expression of *ALKBH5*, *HNRNPC*, *WTAP*, *YTHDF1*, and *YTHDF2* [59]. We found that high m^6^A-related mRNA expression promoted the infiltration of memory B-cells, naïve B-cells, plasma cells, Tregs, monocytes, resting dendritic cells, and eosinophils (Fig. 10, Supplementary Fig. S2). Reducing the level of infiltration of Tregs cells that inhibit the proliferation of CD4+ T cells [65, 66] can effectively improve the OS of cancer patients [67], and a higher level of infiltration of resting DCs is correlated with low OS [68]. In the present study, Treg infiltration levels of Tregs and resting dendritic cells were positively associated with high expression of the four m^6^A-related mRNAs (*AIP*, *CEBPB*, *SDC1* and *VPS25*) (Fig. 10, Supplementary Fig. S2), which also showed high levels of expression in the high-risk CHOL group. These studies indicate that infiltration with Tregs and resting dendritic cells, which is promoted by the four m^6^A-related mRNAs, leads to the adverse outcomes observed in the high m^6^A-related mRNA risk group. This conclusion requires further verification in large-scale experiments.

## Conclusions

In summary, our results provide evidence for the predictive prognosis of CHOL and demonstrated that TMB is not the only prognostic model that can be used to predict the prognosis for patients with CHOL. The m^6^A-related mRNA risk predictive model shows promise for screening patients with CHOL with better immunotherapy response.

## Supplementary Information

**Additional file 1: Supplementary Table S1.** m6A-related mRNA set (*n =* 1281).

**Additional file 2: Supplementary Fig. S1.** Survival analysis of CHOL patients with m6A-related mRNA expression. **A**-**E**) Kaplan–Meier survival of *AIP* (**A**), *CEBPB* (**B**), *SDC1* (**C**), *VPS23* (**D**) and *STXBP2* (**E**) in CHOL patient. CHOL: Cholangiocarcinoma; *AIP*: Aryl hydrocarbon receptor interacting protein; *CEBPB*: CCAAT/enhancer binding protein beta; *SDC1*:syndecan1; *VPS25*: vacuolar protein sorting 25 homolog; *STXBP2*: syntaxin binding protein 2.

**Additional file 3: Supplementary Table S2.** Candidata target genes of CCAAT/enhancer binding protein beta (*n =* 1742)

**Additional file 4: Supplementary Table S3.** Comparison of 22 tumor-infiltrating immune cell types between the low- and high-risk groups.

**Additional file 5: Supplementary Table S4.** Comparison of 22 tumor-infiltrating immune cell types between the low- and high-risk groups.

**Additional file 6: Supplementary Fig. S2.** Correlation between the proportion of tumor-infiltrating cells in the two risk groups with expression of the five m6A-related mRNAs. **A**) Scatterplot of the relationship between the abundance of the 22 TICs and *STXBP2* expression. **B**) Scatterplot of the relationship between the abundance of the 22 TICs and *CEBPB* expression. **C**) Scatterplot of the relationship between the abundance of 22 TICs and *VPS25* expression. *CEBPB*: CCAAT/enhancer binding protein beta; *VPS25*: vacuolar protein sorting 25 homolog; *STXBP2*: syntaxin binding protein 2.

## Data Availability

The datasets GSE89748 and GSE107943 for this study can be found in the GEO database (https://www.ncbi.nlm.nih.gov/geo/). The datasets TCGA of CHOL for this study can be found in the TCGA project (https://portal.gdc.cancer.gov/).

## Abbreviations

CHOL: Cholangiocarcinoma
OS: Overall survival
AUC: Area under curve
ROC: Receiver operating characteristics
MRMRPM: Five m^6^A-related mRNAs risk prognostic model
CCLE: Cancer Cell Line Encyclopedia
HR: Hazard ratio
IME: Immune microenvironment
*AIP*: Aryl hydrocarbon receptor interacting protein
*CEBPB*: CCAAT/enhancer binding protein beta
*SDC1*: Syndecan1
*VPS25*: Vacuolar protein sorting 25 homolog
*STXBP2*: Syntaxin Binding Protein 2
TCGA: The Cancer Genome Atlas
GO: Gene Ontology
KEGG: Kyoto Encyclopedia of Genes and Genomes
PPI: Protein-protein interaction
LASSO: Least absolute shrinkage and selection operator
GSE: Gene set enrichment
GEO: Gene Expression Omnibus
PCA: Principal component analysis
TMB: Tumor mutational burden
DC: Endritic cell
Tregs: T cells regulatory
ESCRT: Essential endocytoid sorting complex
TICs: Tumor-infiltrating immune cells
NK: Natural killer
*METTL*: Methyltransferase-like proteins
*SOCS2*: Suppressor of cytokine signaling 2
