# Supplementary material for: Potential prognosis index for m6A-related mRNA in cholangiocarcinoma
Source: BMC Cancer. 2022 Jun 7;22:620. doi: 10.1186/s12885-022-09665-3 (PMC9170563; doi:10.1186/s12885-022-09665-3)
Supplement: Supplementary file 1 — Additional file 1: Supplementary Table S1. m6A-related mRNA set (n = 1281). [file 12885_2022_9665_MOESM1_ESM.doc]

Supplementary Table S1. m6A-related mRNA set (n = 1281).

| m^6^A-related mRNAs | | | | | | | | |
| --- | --- | --- | --- | --- | --- | --- | --- | --- |
| *AACS* | *BRMS1* | *CTNNBL1* | *FBL* | *ID4* | *MRPS33* | *PHPT1* | *RGL3* | *SLC29A2* |
| *AATF* | *BST2* | *CTNND2* | *FBLN1* | *IFI30* | *MRPS7* | *PHYH* | *RGS4* | *SLC2A4RG* |
| *ABCA2* | *BTBD2* | *CTSA* | *FBXL15* | *IGBP1* | *MRRF* | *PI4K2B* | *RHOBTB2* | *SLC2A6* |
| *ABHD14A* | *BTN2A2* | *CTSW* | *FBXO10* | *IL27RA* | *MSRB2* | *PIGU* | *RHOF* | *SLC2A8* |
| *ABHD14B* | *BTN3A1* | *CTU2* | *FBXO17* | *ILVBL* | *MT1E* | *PIH1D1* | *RHOG* | *SLC38A10* |
| *ABHD8* | *BTN3A2* | *CTXN1* | *FBXO44* | *INHBB* | *MT1F* | *PIN1* | *RHOU* | *SLC39A11* |
| *ACACB* | *BTN3A3* | *CUEDC2* | *FBXW5* | *INO80C* | *MT1M* | *PIN4* | *RILPL1* | *SLC39A7* |
| *ACADS* | *BUD13* | *CUTA* | *FBXW9* | *INO80E* | *MTMR14* | *PIPOX* | *RNASEH2C* | *SLC6A1* |
| *ACBD4* | *C11orf68* | *CWC15* | *FCGRT* | *INSL3* | *MTR* | *PKIB* | *RNF10* | *SLC6A12* |
| *ACD* | *C12orf43* | *CWF19L1* | *FDX1* | *IPO4* | *MVD* | *PKIG* | *RNF126* | *SLC6A16* |
| *ACMSD* | *C12orf45* | *CXXC1* | *FGB* | *IQCD* | *MVK* | *PKLR* | *RNF130* | *SLCO1B1* |
| *ACOT7* | *C12orf57* | *CYB5A* | *FGF2* | *ISCU* | *MVP* | *PKN1* | *RNF187* | *SLFN12* |
| *ACOT8* | *C12orf76* | *CYB5R3* | *FGFBP3* | *ISG15* | *MYL12A* | *PLA2G15* | *RNF216* | *SLFN13* |
| *ACOX3* | *C14orf93* | *CYBA* | *FGG* | *ITGB1BP1* | *MYL5* | *PLEKHJ1* | *RNF26* | *SLPI* |
| *ACSL1* | *C16orf74* | *CYC1* | *FGL1* | *JAM2* | *MYL9* | *PLLP* | *RNF5* | *SMPD1* |
| *ACSM1* | *C18orf21* | *CYCS* | *FHOD1* | *JMJD4* | *NAGLU* | *PLXNB2* | *ROMO1* | *SNAP47* |
| *ACSM2A* | *C19orf24* | *CYGB* | *FKBP8* | *JUND* | *NAGS* | *PMF1* | *RPF1* | *SNAPC4* |
| *ACSM2B* | *C19orf25* | *CYP21A2* | *FKBPL* | *KAAG1* | *NANS* | *PMM1* | *RPL10A* | *SNRNP35* |
| *ACTG1* | *C19orf53* | *CYP2B6* | *FLOT1* | *KANK1* | *NAP1L2* | *PMPCA* | *RPL11* | *SNRNP70* |
| *ACTR1A* | *C1orf50* | *CYP2C8* | *FMO3* | *KANK3* | *NARF* | *PNPO* | *RPL12* | *SNRPA* |
| *ACY3* | *C1R* | *D2HGDH* | *FNDC4* | *KCNK7* | *NAT9* | *POLA2* | *RPL13* | *SNRPC* |
| *ADAM11* | *C2* | *DAND5* | *FOXRED1* | *KCTD17* | *NCAPH2* | *POLD2* | *RPL13A* | *SNRPD1* |
| *ADAMTS13* | *C2CD4C* | *DAP* | *FPGS* | *KHK* | *NCLN* | *POLL* | *RPL14* | *SNRPD2* |
| *ADAP1* | *C4B* | *DAPK1* | *FRG1* | *KIAA1586* | *NDUFA11* | *POLM* | *RPL17* | *SNX17* |
| *ADM* | *C4BPA* | *DAPK3* | *FSCN1* | *KIF12* | *NDUFA13* | *POLR2E* | *RPL18* | *SORBS3* |
| *ADRM1* | *C4orf48* | *DBNL* | *FSCN2* | *KIFC3* | *NDUFA4* | *POLR2I* | *RPL18A* | *SOX18* |
| *AFM* | *C5* | *DBP* | *FSTL3* | *KISS1* | *NDUFA6* | *POLR3H* | *RPL19* | *SP100* |
| *AFMID* | *C6* | *DCDC2* | *FTL* | *KLC3* | *NDUFA7* | *POMP* | *RPL21* | *SP140L* |
| *AGMAT* | *C6orf48* | *DCTN3* | *FUT6* | *KLHDC4* | *NDUFA9* | *POP5* | *RPL23* | *SPATA2L* |
| *AGPAT1* | *C6orf62* | *DCTPP1* | *FXN* | *KLHDC7B* | *NDUFAB1* | *PPAN* | *RPL23A* | *SPATC1* |
| *AGPAT2* | *C7orf26* | *DCXR* | *FXYD2* | *KLKB1* | *NDUFB11* | *PPARGC1A* | *RPL27* | *SPIN2B* |
| *AGT* | *C7orf50* | *DDA1* | *G0S2* | *KPNA2* | *NDUFB2* | *PPDPF* | *RPL27A* | *SPPL3* |
| *AGXT* | *C8G* | *DDR1* | *G6PC* | *KRT10* | *NDUFB6* | *PPFIA3* | *RPL29* | *SS18* |
| *AIF1* | *C9orf116* | *DDX19B* | *G6PC3* | *KRT19* | *NDUFB7* | *PPIA* | *RPL3* | *SSBP1* |
| *AIMP2* | *C9orf16* | *DDX49* | *GAA* | *KRT23* | *NDUFB8* | *PPIE* | *RPL32* | *SSBP4* |
| *AIP* | *CADM4* | *DDX51* | *GADD45GIP1* | *LANCL2* | *NDUFC1* | *PPIF* | *RPL34* | *SSNA1* |
| *AK1* | *CALR* | *DDX56* | *GALE* | *LBP* | *NDUFC2* | *PPIH* | *RPL35* | *SSSCA1* |
| *AKAP8L* | *CAMK1D* | *DEXI* | *GALK1* | *LBR* | *NDUFS4* | *PPIL3* | *RPL35A* | *ST3GAL3* |
| *AKR1A1* | *CANX* | *DHDDS* | *GAMT* | *LCAT* | *NDUFS6* | *PPM1G* | *RPL36* | *STAP2* |
| *AKR1C3* | *CAPN1* | *DHPS* | *GAPDH* | *LCMT1* | *NDUFS7* | *PPP1CA* | *RPL36AL* | *STARD3* |
| *AKR1C4* | *CAPNS1* | *DHRS1* | *GARS* | *LDHD* | *NDUFS8* | *PPP1R3C* | *RPL37* | *STMN1* |
| *ALDH16A1* | *CAPS* | *DHRS11* | *GATC* | *LGALS2* | *NDUFV1* | *PPP2R1B* | *RPL38* | *STOM* |
| *ALDOB* | *CARD11* | *DHRS4L2* | *GCAT* | *LGALS3BP* | *NDUFV2* | *PPP3CC* | *RPL6* | *STT3A* |
| *ALKBH2* | *CARD9* | *DMAP1* | *GCH1* | *LGALS9* | *NECAB3* | *PPP4C* | *RPL7A* | *STX10* |
| *ALKBH7* | *CASP10* | *DMPK* | *GDF15* | *LHB* | *NEDD8* | *PPP5C* | *RPL8* | *STX4* |
| *AMBP* | *CASP8* | *DNAH14* | *GDF7* | *LIG1* | *NEU1* | *PQLC1* | *RPL9* | *STXBP2* |
| *AMH* | *CBY1* | *DNAJB12* | *GEMIN7* | *LIMCH1* | *NFKB2* | *PRAM1* | *RPLP0* | *SULT1A1* |
| *AMPD3* | *CCDC102A* | *DNAJC1* | *GFOD2* | *LIME1* | *NFKBIB* | *PRDX4* | *RPP21* | *SUPV3L1* |
| *AMT* | *CCDC12* | *DNAJC8* | *GGA2* | *LIN7B* | *NFKBIL1* | *PRDX6* | *RPP38* | *SURF1* |
| *AMZ2* | *CCDC124* | *DNASE2* | *GIPC1* | *LIPG* | *NIF3L1* | *PRKAR1B* | *RPS10* | *SURF2* |
| *ANAPC11* | *CCDC137* | *DNHD1* | *GIPR* | *LMF2* | *NINJ1* | *PRKCSH* | *RPS11* | *SYCE1L* |
| *ANAPC4* | *CCDC149* | *DOHH* | *GKAP1* | *LONP1* | *NMT2* | *PRMT1* | *RPS12* | *SYNGR1* |
| *ANG* | *CCDC57* | *DOK4* | *GLDC* | *LPAR2* | *NNT* | *PRMT7* | *RPS13* | *SYNGR2* |
| *ANGPTL3* | *CCDC85B* | *DOK7* | *GLIS3* | *LPCAT1* | *NOC4L* | *PROC* | *RPS15* | *TAF10* |
| *ANGPTL4* | *CCDC92* | *DPM2* | *GMDS* | *LRFN1* | *NOP58* | *PROCA1* | *RPS15A* | *TAF13* |
| *ANKRD13D* | *CCDC96* | *DPM3* | *GNAI3* | *LRG1* | *NOSIP* | *PRODH2* | *RPS16* | *TAPBP* |
| *ANKRD29* | *CCL15* | *DPP7* | *GNG5* | *LRPAP1* | *NOXA1* | *PRR22* | *RPS18* | *TARBP1* |
| *ANO7* | *CCL2* | *DTNA* | *GNMT* | *LRRC20* | *NPAS1* | *PRR5* | *RPS19* | *TARS* |
| *ANO8* | *CCM2* | *DTNBP1* | *GOT1* | *LRRC29* | *NPDC1* | *PRRG2* | *RPS19BP1* | *TAT* |
| *ANXA8* | *CCNB1IP1* | *DTYMK* | *GOT2* | *LRRC45* | *NPEPL1* | *PRSS23* | *RPS20* | *TBC1D22A* |
| *AP1M1* | *CCT3* | *DUS1L* | *GPATCH3* | *LRRC46* | *NPM2* | *PRSS8* | *RPS21* | *TBCC* |
| *AP2S1* | *CCT6B* | *DUS3L* | *GPLD1* | *LRSAM1* | *NPM3* | *PSAT1* | *RPS24* | *TBRG4* |
| *APBA3* | *CD177* | *DUSP10* | *GPR162* | *LSM2* | *NPTX1* | *PSENEN* | *RPS25* | *TCAP* |
| *APOBEC3C* | *CD2BP2* | *DUSP28* | *GPRC5C* | *LSM3* | *NR0B2* | *PSMA1* | *RPS28* | *TCEA2* |
| *APOBEC3D* | *CD320* | *DYNLL1* | *GPRIN2* | *LSM4* | *NR2C2AP* | *PSMA2* | *RPS29* | *TCEA3* |
| *APOBEC3F* | *CD40* | *DYNLRB1* | *GPS1* | *LSM7* | *NR2F2* | *PSMA5* | *RPS3A* | *TCTEX1D4* |
| *APOBEC3G* | *CD58* | *DYNLT1* | *GPSM1* | *LSR* | *NR2F6* | *PSMA7* | *RPS4X* | *TCTN2* |
| *APOC1* | *CD81* | *DYRK1B* | *GPT* | *LST1* | *NRG1* | *PSMB1* | *RPS6* | *TDO2* |
| *APOC2* | *CDC34* | *DYRK4* | *GPT2* | *LTBR* | *NRGN* | *PSMB10* | *RPS6KB2* | *TFB2M* |
| *APOC3* | *CDC37* | *EBNA1BP2* | *GPX1* | *LY6G5C* | *NSMCE1* | *PSMB3* | *RPS7* | *THAP4* |
| *APOE* | *CDC42EP1* | *ECH1* | *GPX4* | *LYPD6B* | *NSUN6* | *PSMB7* | *RPS8* | *THYN1* |
| *APOH* | *CDH6* | *ECSIT* | *GPX8* | *LYPLA2* | *NT5C* | *PSMB8* | *RPSA* | *TIGD1* |
| *APOL1* | *CDK2AP2* | *EDA* | *GRB7* | *LYRM4* | *NUBP1* | *PSMB9* | *RRAS* | *TIMM13* |
| *APOL2* | *CDK5RAP1* | *EDF1* | *GRN* | *MAD1L1* | *NUCB1* | *PSMC4* | *RUFY1* | *TIMM44* |
| *APOL3* | *CDK9* | *EEF1A2* | *GRPEL1* | *MAMDC4* | *NUDC* | *PSMC5* | *RUVBL2* | *TIMM50* |
| *APOL6* | *CDKN1A* | *EEF1B2* | *GTF3A* | *MAN2A2* | *NUDT1* | *PSMD14* | *S1PR2* | *TIMM8B* |
| *APOM* | *CEBPA* | *EEF1E1* | *GTF3C5* | *MANBAL* | *NUDT13* | *PSMD8* | *SAC3D1* | *TK2* |
| *APRT* | *CEBPB* | *EEPD1* | *GTPBP3* | *MAP1LC3A* | *NUDT18* | *PSMD9* | *SAMD1* | *TM7SF3* |
| *AQP1* | *CFB* | *EFCAB6* | *GUCA2B* | *MAP1S* | *NUP210* | *PSME1* | *SAMM50* | *TMED1* |
| *AQP7* | *CFD* | *EGFL7* | *GZMA* | *MAP2K2* | *NUPL2* | *PSME2* | *SARDH* | *TMEM101* |
| *ARFGAP1* | *CFDP1* | *EI24* | *H2AFJ* | *MAP3K11* | *NUPR1* | *PSMG2* | *SARS* | *TMEM104* |
| *ARFRP1* | *CFL1* | *EID2B* | *H2AFX* | *MAPK11* | *NUTF2* | *PSMG3* | *SCAF1* | *TMEM120B* |
| *ARG1* | *CHCHD1* | *EIF1* | *H2AFY2* | *MAPK12* | *NXNL2* | *PSMG4* | *SCAND1* | *TMEM125* |
| *ARHGDIA* | *CHCHD2* | *EIF3B* | *H2AFZ* | *MARS2* | *OAS1* | *PSORS1C1* | *SCLY* | *TMEM126B* |
| *ARHGEF16* | *CHEK2* | *EIF3F* | *HAMP* | *MAST3* | *OASL* | *PSPH* | *SCN1B* | *TMEM129* |
| *ARHGEF18* | *CHMP1A* | *EIF3G* | *HCCS* | *MAT1A* | *OAZ1* | *PSPN* | *SCO2* | *TMEM134* |
| *ARL16* | *CHMP4A* | *EIF3K* | *HCN2* | *MATK* | *OCEL1* | *PTGES2* | *SCRN2* | *TMEM141* |
| *ARL2* | *CHMP4B* | *EIF4EBP1* | *HDAC10* | *MBD3* | *OCIAD2* | *PTMS* | *SCYL1* | *TMEM147* |
| *ARL4D* | *CHMP6* | *EIF6* | *HES4* | *MCAT* | *ODF3B* | *PTOV1* | *SDC1* | *TMEM14C* |
| *ARL6IP4* | *CHMP7* | *ELAC1* | *HIF1AN* | *MCM5* | *OGDHL* | *PUS1* | *SDC4* | *TMEM151A* |
| *ARNTL* | *CHST12* | *ELFN2* | *HIGD1A* | *MDM4* | *OGFR* | *PXMP2* | *SDF2* | *TMEM160* |
| *ARPC3* | *CHST4* | *ELMO3* | *HINT2* | *ME3* | *OLFML3* | *PYCARD* | *SDHAF1* | *TMEM184A* |
| *ARPC4* | *CHST7* | *ELOF1* | *HIST1H1C* | *MECR* | *OPTN* | *PYGL* | *SDHB* | *TMEM184B* |
| *ARPC5L* | *CIRBP* | *ELOVL1* | *HIST1H2AE* | *MED18* | *ORM1* | *QDPR* | *SDHD* | *TMEM203* |
| *ARRDC1* | *CISD3* | *ELOVL5* | *HIST1H4E* | *MED8* | *ORM2* | *QPCTL* | *SDS* | *TMEM205* |
| *ARSA* | *CKB* | *ENDOD1* | *HIST2H2AC* | *METRNL* | *ORMDL3* | *QPRT* | *SDSL* | *TMEM208* |
| *ARV1* | *CKLF* | *ENDOG* | *HLA-A* | *MFAP2* | *OSGEPL1* | *QTRT1* | *SEC61B* | *BPHL* |
| *ASNA1* | *CLEC14A* | *ENHO* | *HLA-B* | *MGAT4B* | *P2RX7* | *R3HCC1* | *SEC61G* | *CTDP1* |
| *ASPHD1* | *CLIC1* | *ENO1* | *HLA-C* | *MIA3* | *P2RY11* | *RAB1B* | *SECTM1* | *FAU* |
| *ASS1* | *CLN3* | *ENTPD8* | *HLA-DMA* | *MLF2* | *P4HB* | *RAB20* | *SEH1L* | *ICAM1* |
| *ATF4* | *CLPP* | *EPB41L1* | *HLA-DRB1* | *MLLT3* | *PABPN1* | *RAB3A* | *SELP* | *MRPS25* |
| *ATP13A1* | *CLPTM1* | *EPB41L3* | *HLA-E* | *MMAB* | *PACSIN2* | *RAB4B* | *SEMA4G* | *PHKG2* |
| *ATP1A1* | *CLPTM1L* | *ERCC1* | *HLA-G* | *MMP9* | *PAEP* | *RAB5C* | *SEPHS2* | *RFXANK* |
| *ATP6V0B* | *CLU* | *ERI3* | *HLX* | *MOCOS* | *PAFAH1B3* | *RABAC1* | *SERINC2* | *SLC27A4* |
| *ATP6V0D1* | *CMTM3* | *ERP29* | *HMBOX1* | *MORN4* | *PAQR6* | *RABGGTA* | *SERPINB6* | *BOLA1* |
| *B3GNT3* | *CMTM8* | *ETNK2* | *HMBS* | *MPND* | *PAQR7* | *RAC3* | *SERPINC1* | *CSDE1* |
| *B4GALT2* | *CNPY3* | *ETV2* | *HMG20B* | *MPST* | *PARP3* | *RAD9A* | *SESN2* | *FARSA* |
| *B9D2* | *COASY* | *EXD3* | *HMGCL* | *MPV17L2* | *PAX8* | *RALY* | *SF3B5* | *IAH1* |
| *BAIAP2* | *COMMD3* | *EXOC8* | *HMOX1* | *MRC1* | *PCSK4* | *RAMP1* | *SGTA* | *MRPS18B* |
| *BAK1* | *COMMD6* | *EXOSC1* | *HOMER3* | *MRM1* | *PCYT2* | *RAMP2* | *SH2D3A* | *PHB2* |
| *BANF1* | *COMTD1* | *EXOSC4* | *HP* | *MRPL11* | *PDE5A* | *RAN* | *SHARPIN* | *RFNG* |
| *BAX* | *COPE* | *EXOSC5* | *HPN* | *MRPL12* | *PDF* | *RANBP10* | *SHB* | *SLC25A39* |
| *BCAM* | *COX4I1* | *F11* | *HPS1* | *MRPL17* | *PDGFD* | *RANBP3* | *SHC2* | *BOLA2B* |
| *BCAN* | *COX6A1* | *FABP3* | *HPX* | *MRPL33* | *PDLIM1* | *RARA* | *SIRT2* | *CSNK2B* |
| *BCAR1* | *COX6B1* | *FAM118B* | *HS1BP3* | *MRPL34* | *PDXP* | *RASIP1* | *SIRT6* | *FASN* |
| *BCAS2* | *CPLX1* | *FAM160B2* | *HSBP1* | *MRPL36* | *PDZD11* | *RBBP5* | *SKIV2L* | *ICA1L* |
| *BCAS4* | *CPNE1* | *FAM166B* | *HSCB* | *MRPL39* | *PDZK1IP1* | *RBM34* | *SLC12A7* | *MRPS24* |
| *BCKDK* | *CPNE2* | *FAM171A1* | *HSD11B1L* | *MRPL4* | *PEF1* | *RBM42* | *SLC17A2* | *PHGDH* |
| *BCL2* | *CPPED1* | *FAM174B* | *HSD3B7* | *MRPL41* | *PEPD* | *RBP4* | *SLC19A3* | *RFX5* |
| *BCL7C* | *CPT1C* | *FAM177B* | *HSP90B1* | *MRPL48* | *PEX11G* | *RBP5* | *SLC22A1* | *SLC25A42* |
| *BDH1* | *CRB3* | *FAM20C* | *HSPB6* | *MRPL51* | *PEX6* | *RBPMS* | *SLC22A18* |  |
| *BICC1* | *CREB3L3* | *FAM32A* | *HSPB8* | *MRPL52* | *PFDN4* | *RBX1* | *SLC22A3* |  |
| *BIN1* | *CREB5* | *FAM89B* | *HTRA3* | *MRPL54* | *PFDN6* | *REEP4* | *SLC25A10* |  |
| *BLOC1S2* | *CRELD2* | *FAM98C* | *HUS1* | *MRPL55* | *PFKP* | *REEP6* | *SLC25A16* |  |
| *BMP1* | *CRYL1* | *FANCC* | *HYI* | *MRPS12* | *PGM1* | *RELA* | *SLC25A23* |  |
| *BMP4* | *CRYM* | *FARS2* | *HYOU1* | *MRPS17* | *PGPEP1* | *RERG* | *SLC25A28* |  |
