# Supplementary material for: Potential prognosis index for m6A-related mRNA in cholangiocarcinoma
Source: BMC Cancer. 2022 Jun 7;22:620. doi: 10.1186/s12885-022-09665-3 (PMC9170563; doi:10.1186/s12885-022-09665-3)
Supplement: Supplementary file 3 — Additional file 3: Supplementary Table S2. Candidata target genes of CCAAT/enhancer binding protein beta (n = 1742) [file 12885_2022_9665_MOESM3_ESM.docx]

Supplementary Table S2. Candidata target genes of CEBPB (n = 1742)

| Candidata target genes of CEBPB | | | | | | | | |
| --- | --- | --- | --- | --- | --- | --- | --- | --- |
| AAAS | AASS | ABCA17P | ABCA2 | ABCC3 | ABCC5 | ABCF2 | ABHD15 | ABHD3 |
| ABHD5 | ABTB2 | ACAA1 | ACAT2 | ACBD4 | ACHE | ACKR1 | ACN9 | ACOT4 |
| ACOXL | ACP2 | ACTA2 | ACTL10 | ACTR3 | ACY1 | ADAM1B | ADAM28 | ADAMTS6 |
| ADAMTS7 | ADAMTSL5 | ADCK3 | ADCK4 | ADCY9 | ADH4 | ADIPOQ | ADIPOR1 | ADORA2B |
| ADPRHL2 | ADRB2 | ADRBK1 | AES | AGBL3 | AGBL5 | AGER | AGPAT1 | AGPAT5 |
| AGTRAP | AHNAK | AIM2 | AIP | AK4 | AKNA | AKR1D1 | ALDH3B1 | ALDH6A1 |
| ALKBH5 | ALLC | ALOX12 | ALOXE3 | ALPK3 | AMBP | AMD1 | AMER2 | AMT |
| AMZ2 | ANAPC1 | ANAPC11 | ANK1 | ANK2 | ANKRD34C | ANKRD40 | ANKRD42 | ANKRD45 |
| ANKRD49 | ANKRD52 | ANKRD6 | ANLN | ANO6 | ANP32E | ANXA3 | ANXA6 | ANXA9 |
| AP2B1 | AP2M1 | AP5S1 | APBA3 | APMAP | APOB | APOBEC1 | APOF | APP |
| APPBP2 | AQP12A | AQP3 | AQP8 | AQP9 | AREL1 | ARF5 | ARHGAP26 | ARHGAP27 |
| ARHGEF11 | ARHGEF2 | ARHGEF28 | ARHGEF33 | ARHGEF37 | ARID3A | ARMC1 | ARMC8 | ASAP1 |
| ASGR2 | ASH1L | ASPG | ASXL2 | ATAD5 | ATCAY | ATF3 | ATF4 | ATG16L1 |
| ATG4D | ATMIN | ATOH7 | ATP11A | ATP13A2 | ATP13A3 | ATP2B1 | ATP6V1C1 | ATP6V1F |
| ATP8A2 | ATPIF1 | AURKB | AVPI1 | AXDND1 | AZGP1 | AZIN1 | B3GNT1 | B3GNT3 |
| B4GALT3 | B4GALT6 | B9D1 | BACE1 | BACE2 | BAD | BAHD1 | BAIAP2 | BAIAP2L1 |
| BATF2 | BATF3 | BBS5 | BCCIP | BCL2L1 | BCL2L10 | BCL2L13 | BCL6 | BCL6B |
| BCO2 | BECN1 | BEST1 | BGLAP | BHLHE23 | BHLHE41 | BIN1 | BLOC1S4 | BMF |
| BRD2 | BRF2 | BTBD17 | BTBD2 | BTN1A1 | BTN2A2 | BUD31 | BZRAP1 | C10orf53 |
| C10orf62 | C11orf57 | C12orf57 | C15orf39 | C15orf48 | C15orf59 | C16orf86 | C17orf107 | C17orf49 |
| C17orf59 | C17orf62 | C17orf66 | C17orf72 | C17orf85 | C19orf24 | C19orf43 | C19orf44 | C19orf59 |
| C1orf109 | C1orf115 | C1orf123 | C1orf127 | C1orf64 | C1QTNF1 | C1QTNF4 | C1R | C1RL |
| C1S | C20orf112 | C20orf173 | C2CD4C | C2orf54 | C2orf57 | C3 | C3orf14 | C4B |
| C4orf17 | C4orf36 | C4orf47 | C9 | C9orf169 | C9orf3 | C9orf85 | CACNA1S | CACNA2D3 |
| CACNB1 | CACYBP | CADM3 | CALB2 | CALCA | CAMTA2 | CANT1 | CANX | CAPN11 |
| CAPN2 | CAPRIN2 | CAPZA2 | CARKD | CARM1 | CARNS1 | CARS | CASC1 | CASP1 |
| CASP4 | CASP6 | CATSPER4 | CATSPERG | CBFA2T3 | CBR1 | CBX1 | CBX4 | CBX5 |
| CBX8 | CCDC122 | CCDC158 | CCDC173 | CCDC180 | CCDC22 | CCDC28B | CCDC47 | CCDC51 |
| CCDC69 | CCDC85B | CCDC86 | CCDC88A | CCDC97 | CCL1 | CCL2 | CCL4 | CCL7 |
| CCNB1 | CCNDBP1 | CCNF | CCR3 | CCT4 | CCT7 | CD28 | CD300A | CD300C |
| CD300LB | CD300LD | CD302 | CD320 | CD47 | CD79B | CD9 | CDC25A | CDC25B |
| CDC42SE1 | CDC6 | CDCA2 | CDCA5 | CDH15 | CDH16 | CDH23 | CDH3 | CDK17 |
| CDK2AP2 | CDKN3 | CDNF | CDO1 | CDPF1 | CEBPB | CEBPE | CECR6 | CELA1 |
| CELA2A | CELF5 | CENPU | CEP128 | CEP170 | CES1 | CFAP57 | CFL1 | CGREF1 |
| CHCHD6 | CHD2 | CHEK1 | CHIA | CHPT1 | CHRM3 | CHRNA9 | CHST12 | CHST3 |
| CHST5 | CHURC1 | CIRH1A | CISH | CIZ1 | CKAP2L | CKS2 | CLASP1 | CLCA4 |
| CLCF1 | CLCNKA | CLDN23 | CLEC10A | CLEC12A | CLEC12B | CLEC4D | CLEC4E | CLEC5A |
| CLHC1 | CLIC6 | CLMP | CLN8 | CLP1 | CLPS | CLUAP1 | CLUH | CMAS |
| CMTR1 | CNIH4 | CNOT8 | CNP | CNPPD1 | CNPY2 | CNPY3 | CNR2 | CNTROB |
| COG8 | COL10A1 | COL1A1 | COL23A1 | COL27A1 | COL7A1 | COMMD4 | COMMD5 | COPS4 |
| COQ2 | COQ5 | COQ9 | CORO1B | COTL1 | COX14 | COX15 | COX6A1 | COX7A2 |
| CPB2 | CPEB4 | CPN2 | CPT1B | CREB3L3 | CREB5 | CREG2 | CRYGD | CS |
| CSF2RB | CSGALNACT1 | CSNK1D | CSNK1G1 | CSNK2A2 | CTDNEP1 | CTNNBIP1 | CTRL | CTSK |
| CTU2 | CUEDC2 | CUL2 | CWF19L2 | CXCL16 | CXCR1 | CXCR5 | CXCR6 | CYB561A3 |
| CYP17A1 | CYP51A1 | CYTIP | DARS2 | DAXX | DBNL | DCAF15 | DCLRE1C | DCUN1D3 |
| DDIT3 | DEGS1 | DENND6B | DERL2 | DGAT1 | DGKZ | DHPS | DHRS13 | DHRS2 |
| DHRS7B | DHRS9 | DIO3 | DIRAS2 | DLC1 | DLD | DLEU2 | DLGAP2 | DLGAP4 |
| DLX2 | DMP1 | DNAAF2 | DNAH1 | DNAH8 | DNAJC28 | DNAJC5 | DNAJC7 | DNAL1 |
| DNASE1L2 | DNMT1 | DOCK5 | DOCK8 | DPAGT1 | DPEP2 | DPP3 | DPP8 | DPYS |
| DQX1 | DRAM1 | DRG2 | DSE | DSN1 | DSPP | DTL | DTNBP1 | DTX4 |
| DUOX1 | DUOXA1 | DUSP1 | DUSP12 | DUSP16 | DUSP18 | DUSP28 | DVL2 | DYNLRB1 |
| DYRK3 | DZIP1 | E4F1 | EBAG9 | EBF3 | ECEL1 | ECSCR | EDEM2 | EFEMP2 |
| EFNA2 | EGR2 | EHD1 | EID2B | EIF2AK2 | EIF2AK3 | EIF2AK4 | EIF2S2 | EIF3B |
| EIF3G | EIF3J | EIF4A1 | ELAVL3 | ELMOD3 | ELP4 | EMC10 | EMC7 | EMR4P |
| ENKD1 | ENKUR | ENO3 | ENPP4 | ENTPD6 | ENTPD7 | ENY2 | EPAS1 | EPHB3 |
| EPHB4 | EPM2AIP1 | EPS8L1 | ESYT2 | ETF1 | ETV6 | EVA1B | EVI2A | EVI5L |
| EWSR1 | EXOC6 | EXOSC4 | EXTL1 | F10 | F2 | F7 | F9 | FABP12 |
| FABP5 | FABP9 | FADS1 | FAIM2 | FAM102A | FAM120A | FAM129B | FAM134C | FAM149B1 |
| FAM161B | FAM166B | FAM174A | FAM192A | FAM19A3 | FAM214B | FAM3B | FAM49B | FAM53A |
| FAM71F2 | FAM73B | FAM76A | FAM83F | FAM98A | FANCA | FANCL | FAS | FASLG |
| FBLIM1 | FBXL12 | FBXL21 | FBXL6 | FBXO22 | FBXO24 | FBXO34 | FBXO41 | FBXO47 |
| FBXO48 | FCGR1A | FCGR2B | FCRLA | FDXR | FEN1 | FFAR4 | FGD4 | FGF21 |
| FGFBP3 | FGL2 | FIP1L1 | FKBP15 | FLRT3 | FLYWCH1 | FMNL1 | FMNL2 | FN3K |
| FNBP1L | FNBP4 | FNIP2 | FOSL1 | FOXF1 | FOXJ1 | FOXL2 | FOXO1 | FOXO3 |
| FPGS | FRG1 | FRMD8 | FSCN2 | FXYD4 | FYB | FYCO1 | FZD7 | GAA |
| GABARAP | GABARAPL1 | GABPB2 | GADD45A | GADD45B | GADD45G | GALNT10 | GALNT14 | GAN |
| GANC | GAPDH | GAR1 | GART | GAS2L3 | GAS7 | GATA5 | GBA | GBGT1 |
| GBP2 | GBP4 | GCC1 | GCC2 | GCHFR | GCKR | GCNT1 | GCNT2 | GCNT3 |
| GCNT4 | GEMIN8 | GFM1 | GFPT1 | GFRA1 | GGA1 | GGA3 | GINS1 | GJA10 |
| GLDC | GLTP | GNA13 | GNAI3 | GNAQ | GNAT2 | GNB3 | GNG3 | GNL2 |
| GNL3L | GON4L | GOSR1 | GP5 | GPAA1 | GPBAR1 | GPC3 | GPM6B | GPN2 |
| GPR115 | GPR137C | GPR3 | GPR61 | GPR84 | GPRC5C | GPSM1 | GPT | GRAMD2 |
| GRB2 | GRIN3B | GRINA | GRIP2 | GRIPAP1 | GRN | GSDMA | GSN | GSTK1 |
| GSTM2 | GTF2B | GTF2E2 | GTF2H4 | GTF3C2 | GUCY2C | HAAO | HABP4 | HARS |
| HAUS6 | HAX1 | HCAR2 | HCFC1R1 | HCRT | HDHD3 | HES5 | HES7 | HEXIM2 |
| HFE2 | HGF | HHEX | HIAT1 | HIST1H3B | HIST1H3G | HIST2H2BB | HIST2H4A | HJURP |
| HLA-A | HLTF | HM13 | HMBS | HMG20B | HMGB1 | HMGB2 | HMGCS2 | HMGN5 |
| HMHA1 | HMOX1 | HNRNPF | HNRNPH1 | HNRNPK | HNRNPUL2 | HOOK2 | HP | HPGD |
| HPX | HS3ST2 | HSD17B10 | HSD17B2 | HSD17B8 | HSPB8 | HTRA2 | HVCN1 | IARS |
| ICA1L | IDS | IFI30 | IFNA4 | IFRD2 | IFT172 | IFT20 | IFT27 | IFT57 |
| IFT80 | IGF2BP2 | IGFALS | IGFBP7 | IGHMBP2 | IGSF9 | IKZF1 | IL10 | IL10RA |
| IL15RA | IL17B | IL17RE | IL1R1 | IL20RB | IL27RA | IL3 | IL36RN | ING1 |
| ING3 | INPP1 | INPP4B | INPP5K | INSIG2 | INSRR | INTS4 | IP6K3 | IPP |
| IQCC | IQCD | IRAK3 | IRF5 | IRGM | ISG20 | ISLR2 | ITGA7 | ITGA8 |
| ITGB2 | ITIH3 | ITIH4 | ITM2B | ITPKB | ITPRIP | ITPRIPL1 | ITSN1 | IYD |
| IZUMO4 | JAGN1 | JMJD6 | JMJD8 | JPH2 | KANK3 | KAT7 | KAT8 | KCNAB3 |
| KCNE2 | KCNK13 | KCNK5 | KCNN3 | KCTD5 | KDM1B | KDM6B | KDR | KHK |
| KIAA1324 | KIF1B | KLF6 | KLHDC10 | KLHDC9 | KLHL12 | KLHL23 | KLRB1 | KNG1 |
| KNOP1 | KRAS | KXD1 | LAMP1 | LARP1 | LARP4B | LARS | LCP1 | LCP2 |
| LDB1 | LECT2 | LFNG | LGR4 | LILRB3 | LILRB4 | LIME1 | LIN28A | LIN54 |
| LIPC | LMAN1L | LMNB2 | LMO4 | LOC100129361 | LOC102723903 | LONRF3 | LOXL3 | LPXN |
| LRG1 | LRRC16A | LRRC61 | LRRC8C | LRRK1 | LTA4H | LTBP4 | LUC7L | LY6E |
| LYN | LYRM7 | LYRM9 | LYST | LYZ | LZIC | LZTFL1 | MAEA | MAGEH1 |
| MAGOHB | MALAT1 | MAML1 | MAN1B1 | MAN1C1 | MANBAL | MAP10 | MAP1B | MAP3K11 |
| MAP3K14 | MAP3K3 | MAP3K8 | MAP4K2 | MAP4K5 | MAP7D3 | MAPRE3 | MARVELD1 | MARVELD3 |
| MAT1A | MATN3 | MATN4 | MBD2 | MC1R | MCAM | MCIDAS | MDFIC | MDK |
| MDM1 | MDP1 | MED20 | MED30 | MED7 | MED8 | MED9 | MEIS1 | MERTK |
| MET | METRNL | METTL13 | METTL18 | METTL21C | MEX3B | MFSD3 | MFSD5 | MFSD7 |
| MGAT5 | MGRN1 | MIEN1 | MIF4GD | MIP | MIR103A2 | MIR130B | MIR135B | MIR142 |
| MIR185 | MIR186 | MIR21 | MIR22 | MIR29A | MIR379 | MIR499A | MIR504 | MIR511 |
| MIR543 | MIR758 | MIS12 | MIS18BP1 | MKS1 | MLEC | MLLT1 | MLLT11 | MLXIP |
| MMP12 | MMP13 | MMP19 | MMP21 | MMP27 | MMP28 | MMP8 | MNAT1 | MNT |
| MPG | MPHOSPH10 | MPHOSPH9 | MPO | MPZL2 | MRC1 | MRGPRX2 | MRPL11 | MRPL21 |
| MRPL28 | MRPL33 | MRPL35 | MRPL46 | MRPL55 | MRPS10 | MRPS11 | MRPS18A | MRPS36 |
| MRVI1 | MS4A7 | MST1 | MTBP | MTERF2 | MTHFD1L | MTMR11 | MTMR4 | MTMR6 |
| MTO1 | MTRF1L | MTVR2 | MUT | MXD4 | MXI1 | MXRA7 | MYB | MYCBPAP |
| MYD88 | MYL6 | MYL6B | MYO1A | MYO1C | MYO1E | MYO1G | NAA25 | NAE1 |
| NAGLU | NAGS | NAP1L1 | NARS | NARS2 | NASP | NAT10 | NAT6 | NBEAL2 |
| NCF4 | NCKAP1L | NCKIPSD | NCOA6 | NDRG3 | NDRG4 | NDST1 | NDUFA11 | NDUFAF7 |
| NDUFS8 | NEAT1 | NEPNP | NEU2 | NEURL4 | NF1 | NFAT5 | NFE2 | NFKBIL1 |
| NGEF | NHLH1 | NHLH2 | NIFK | NINJ1 | NISCH | NKD2 | NLRP12 | NLRP3 |
| NLRP5 | NME2 | NMNAT1 | NOD1 | NOD2 | NOL7 | NOS2 | NOSTRIN | NOTCH1 |
| NOTCH3 | NPAS2 | NPHS2 | NPL | NPTN | NPY | NQO2 | NR1H3 | NR3C1 |
| NR4A3 | NRG4 | NRN1L | NT5C | NT5C3A | NUAK1 | NUDC | NUDT13 | NUDT15 |
| NUDT21 | NUF2 | NUFIP2 | NUP35 | OCIAD1 | OGFOD2 | OGG1 | OLR1 | OOEP |
| OPA3 | OR10H3 | OR2B6 | OR2D2 | OR4M1 | OR51B2 | OR51E1 | OR51S1 | OR52A5 |
| OR52N4 | OR9A4 | ORM1 | ORM2 | ORMDL2 | OS9 | OSBPL8 | OSCAR | OSM |
| OVOL2 | OXCT1 | OXNAD1 | OXR1 | P2RX3 | P2RY14 | P4HA1 | PABPC1L | PAPLN |
| PAQR7 | PCBD1 | PCBP2 | PCDHGA5 | PCDHGC4 | PCID2 | PCK1 | PCSK1 | PCSK7 |
| PCSK9 | PCYOX1 | PDC | PDCD1LG2 | PDE6H | PDHX | PDIA4 | PDLIM4 | PDRG1 |
| PDS5A | PDXP | PEF1 | PELI1 | PER1 | PEX2 | PEX3 | PFDN5 | PGK1 |
| PGLYRP4 | PHB | PHF12 | PHF14 | PHF23 | PHGDH | PHKG1 | PHLDA1 | PHOSPHO1 |
| PI15 | PI4KA | PIGM | PIGV | PIGW | PIGX | PIK3C2A | PIK3R5 | PIM3 |
| PIP5K1C | PIP5KL1 | PISD | PITPNA | PITPNB | PITPNM1 | PIWIL4 | PLA2G2E | PLA2G3 |
| PLD1 | PLD2 | PLEK | PLEKHF2 | PLEKHG6 | PLEKHH1 | PLEKHM1 | PLEKHO1 | PLXNA1 |
| PM20D1 | PNKD | PNMA3 | PNPLA8 | PNPT1 | POLD4 | POLDIP3 | POLE | POLR1A |
| POLR3GL | POLR3H | POLRMT | POM121 | POMGNT2 | PPA1 | PPAN | PPARGC1B | PPIA |
| PPIL6 | PPIP5K2 | PPM1F | PPME1 | PPP1R11 | PPP1R13B | PPP1R14B | PPP1R14D | PPP1R15B |
| PPP2R3C | PPRC1 | PPT1 | PRCP | PRKAG1 | PRKD3 | PRKX | PRMT3 | PRND |
| PROC | PROSC | PROX2 | PRR11 | PRR13 | PRR15L | PRR23A | PRR3 | PRSS23 |
| PRSS45 | PRTN3 | PSAP | PSMB6 | PSMD9 | PTCD1 | PTGES | PTGIS | PTMS |
| PTP4A1 | PTPN13 | PTPN2 | PTPRO | PTRF | PVRL4 | PXYLP1 | PYGB | PYGO1 |
| QDPR | QTRT1 | QTRTD1 | R3HCC1L | RAB17 | RAB1A | RAB20 | RAB29 | RAB37 |
| RAB3IL1 | RAB42 | RAB43 | RAB4A | RAB7B | RAB8B | RABIF | RAC1 | RAD51D |
| RANBP2 | RAPGEF6 | RAPSN | RASAL2 | RASGRP2 | RAVER1 | RBKS | RBM12B | RBM34 |
| RBM39 | RBM47 | RBP7 | RBPMS2 | RCC1 | RCC2 | RCHY1 | RCN1 | RCN2 |
| RD3 | RDH11 | RECQL5 | REEP5 | RELA | RELL1 | RET | RFX2 | RGS1 |
| RGS19 | RHBDL1 | RHOA | RHOBTB3 | RHOH | RHOT1 | RIOK2 | RLTPR | RND1 |
| RNF166 | RNF180 | RNF20 | RNF24 | RNF26 | RNF40 | RNFT2 | ROCK2 | ROMO1 |
| ROR1 | RPE | RPIA | RPL10A | RPL3 | RPL3L | RPN1 | RPP14 | RPRD1B |
| RPRD2 | RPS14 | RPS19BP1 | RPS24 | RPS6KB2 | RQCD1 | RRP1 | RSG1 | RTN4 |
| RTN4RL1 | RTP2 | RUFY1 | RUFY4 | RUNX1 | RUVBL2 | RXRB | S100A4 | S100PBP |
| S1PR4 | SALL1 | SAMD8 | SAMHD1 | SCARB1 | SCARNA17 | SCARNA6 | SCGB1A1 | SCIMP |
| SCIN | SCN3B | SCO1 | SCOC | SCTR | SDC1 | SDCBP | SDHAF2 | SDHD |
| SDK2 | SDR42E1 | SEC14L5 | SEC16A | SECISBP2 | SELL | SEMA3B | SEMA3F | SEMA3G |
| SEMG1 | SENP2 | SENP3 | SEPP1 | SEPT8 | SERPINA12 | SERPINA3 | SERPINA9 | SERPINB2 |
| SERPINB5 | SERPINB7 | SERPINC1 | SERPINI1 | SERTM1 | SET | SETD5 | SETD8 | SETDB1 |
| SF1 | SF3B2 | SF3B4 | SF3B5 | SFPQ | SGSH | SGTB | SH2D2A | SH3BP5L |
| SH3GL3 | SH3PXD2B | SHD | SHISA5 | SHQ1 | SIGLEC1 | SIL1 | SIN3B | SIPA1 |
| SIPA1L3 | SKI | SLAMF9 | SLBP | SLC10A1 | SLC12A6 | SLC16A10 | SLC16A7 | SLC1A2 |
| SLC1A4 | SLC20A1 | SLC22A7 | SLC23A1 | SLC25A11 | SLC25A24 | SLC25A25 | SLC25A26 | SLC25A45 |
| SLC26A2 | SLC26A6 | SLC29A3 | SLC30A2 | SLC31A1 | SLC34A2 | SLC35A4 | SLC35G2 | SLC35G6 |
| SLC38A10 | SLC38A2 | SLC39A7 | SLC3A2 | SLC46A2 | SLC4A9 | SLC6A18 | SLC6A4 | SLC9A3R1 |
| SLCO2A1 | SLFN12L | SMAD3 | SMAD6 | SMARCA5 | SMARCAD1 | SMARCC2 | SMARCD1 | SMARCD2 |
| SMCR8 | SMG8 | SMPDL3B | SMYD3 | SNAPC2 | SNAPC5 | SNHG11 | SNHG12 | SNHG5 |
| SNORA17 | SNORA21 | SNORD2 | SNORD49B | SNORD64 | SNORD7 | SNORD83B | SNORD87 | SNORD90 |
| SNRPC | SNRPD2 | SNRPE | SNX12 | SNX15 | SNX20 | SNX22 | SOAT1 | SOAT2 |
| SON | SOX6 | SP140 | SPAG4 | SPATA5 | SPATA7 | SPATC1 | SPDL1 | SPIC |
| SPINT4 | SPON1 | SPRY4 | SPTBN1 | SPTY2D1 | SRF | SRFBP1 | SRPK1 | SRPRB |
| SRRM2 | SRSF3 | SRSF5 | SRSF9 | SSB | SSH2 | SSH3 | SSMEM1 | SST |
| ST3GAL4 | ST6GAL1 | ST7 | STAMBPL1 | STAR | STAT4 | STAT6 | STBD1 | STEAP4 |
| STK11 | STK25 | STK36 | STOX2 | STRIP1 | STX11 | STXBP6 | SUV39H1 | SYNC |
| SYNDIG1L | SYNJ2BP | SYNPO2 | SYT11 | SZT2 | TAF15 | TAF1B | TAF1D | TAF5L |
| TAF8 | TAGLN | TALDO1 | TARS | TARS2 | TATDN2 | TATDN3 | TBC1D10C | TBC1D14 |
| TBC1D22A | TBC1D23 | TBC1D5 | TBC1D8 | TBP | TBRG1 | TBX21 | TBXAS1 | TCAM1P |
| TCF12 | TCF25 | TCN2 | TCTEX1D2 | TDGF1 | TDRD7 | TDRD9 | TECPR1 | TEKT3 |
| TEKT4 | TEX14 | TEX33 | TEX43 | TF | TFE3 | TFEB | TFEC | TFRC |
| TGFB2 | TGFBR1 | TGFBR3 | TGIF1 | TGM1 | TGM5 | THBD | THBS1 | THEM4 |
| THOC1 | THRA | THUMPD1 | THUMPD2 | TIGD4 | TIMM22 | TIMM8B | TLR1 | TLX2 |
| TMBIM6 | TMC8 | TMCC1 | TMCC3 | TMCO4 | TMCO6 | TMED7 | TMEM109 | TMEM115 |
| TMEM126B | TMEM134 | TMEM144 | TMEM154 | TMEM189 | TMEM2 | TMEM216 | TMEM229B | TMEM231 |
| TMEM245 | TMEM247 | TMEM25 | TMEM262 | TMEM33 | TMEM52B | TMEM53 | TMEM72 | TMIGD1 |
| TMOD3 | TMPO | TMPRSS7 | TMPRSS9 | TMX4 | TNFAIP3 | TNFAIP6 | TNFAIP8 | TNFRSF11A |
| TNFRSF1A | TNFSF12 | TNFSF13B | TNFSF15 | TNIP1 | TNIP2 | TNNC1 | TNRC6B | TNXB |
| TOMM20 | TOMM20L | TOMM6 | TOR4A | TP53 | TP53BP2 | TP53INP2 | TPD52L3 | TPPP |
| TPRN | TPSB2 | TPT1P1 | TRA2A | TRAPPC4 | TREH | TREM2 | TREX2 | TRIAP1 |
| TRIB1 | TRIB3 | TRIM14 | TRIM16 | TRIM39 | TRIM46 | TRIM56 | TRIM75P | TRIM8 |
| TRIO | TRMT10A | TRMT12 | TRMT44 | TRMU | TRPV2 | TSPO | TSPY26P | TSPYL2 |
| TST | TTC19 | TTC24 | TTC26 | TTC33 | TTC36 | TTC39A | TTC39C | TTC9C |
| TTI2 | TTL | TTLL1 | TTLL13 | TUBA1A | TUBA1B | TUBA1C | TUBD1 | TULP4 |
| TWF2 | TXLNA | TXNRD3 | UBASH3B | UBB | UBE2N | UBE2V1 | UBE4B | UBL3 |
| UBR4 | UBTD1 | UBXN1 | UCK2 | UGP2 | UGT1A1 | UGT1A10 | UGT1A9 | UGT2B10 |
| UGT3A2 | UNC45A | UNC45B | UNC5A | UNC93B1 | UNK | UOX | UQCC1 | UQCR10 |
| UQCRFS1 | URAD | UROS | USF1 | USP2 | USP21 | USP27X | USP48 | VAMP5 |
| VANGL1 | VASH2 | VASN | VEPH1 | VIM | VMP1 | VNN3 | VOPP1 | VPS11 |
| VPS28 | VPS35 | VPS37C | VSIG8 | VTN | VWA3A | VWF | WBP1L | WDPCP |
| WDR11 | WDR18 | WDR19 | WDR24 | WDR31 | WDR41 | WDR70 | WDR90 | WDR92 |
| WDSUB1 | WEE1 | WFDC11 | WIBG | WIPF2 | XBP1 | XIST | XKR7 | XPA |
| XPO5 | XRCC5 | XRCC6 | YBX2 | YTHDF1 | ZAP70 | ZBTB1 | ZBTB25 | ZBTB48 |
| ZBTB8OS | ZC3H14 | ZDHHC18 | ZDHHC5 | ZER1 | ZFP1 | ZFP14 | ZFPM1 | ZFR2 |
| ZFYVE26 | ZHX1 | ZHX2 | ZKSCAN3 | ZMAT5 | ZMYND8 | ZNF174 | ZNF18 | ZNF205 |
| ZNF212 | ZNF213 | ZNF219 | ZNF226 | ZNF316 | ZNF384 | ZNF420 | ZNF616 | ZNF627 |
| ZNF664 | ZNF672 | ZNF729 | ZNF746 | ZNHIT6 | ZNRF2 | ZP3 | ZRANB3 | ZRSR1 |
| ZSCAN10 | ZSCAN30 | ZUFSP | ZZEF1 | ZZZ3 |  |  |  |  |
