# Supplementary material for: Potential prognosis index for m6A-related mRNA in cholangiocarcinoma
Source: BMC Cancer. 2022 Jun 7;22:620. doi: 10.1186/s12885-022-09665-3 (PMC9170563; doi:10.1186/s12885-022-09665-3)
Supplement: Supplementary file 4 — Additional file 4: Supplementary Table S3. Comparison of 22 tumor-infiltrating immune cell types between the low- and high-risk groups. [file 12885_2022_9665_MOESM4_ESM.doc]

Supplementary Table S3. Identification of drug sensitivity using the m6A-related mRNA model.

| Drug | P value | Drug | P value | Drug | P value | Drug | P value |
| --- | --- | --- | --- | --- | --- | --- | --- |
| A.443654 | 0.95 | Bexarotene | 0.87 | CHIR.99021 | 0.53 | Gemcitabine | 0.75 |
| A.770041 | 0.19 | BI.2536 | 0.89 | CI.1040 | 0.69 | GNF.2 | 0.29 |
| ABT.263 | 0.94 | BIBW2992 | 0.65 | Cisplatin | 0.75 | GSK269962A | 0.95 |
| ABT.888 | 0.79 | Bicalutamide | 0.56 | CMK | 0.72 | GSK.650394 | 0.78 |
| AG.014699 | 0.78 | BI.D1870 | 0.51 | Cyclopamine | 0.48 | GW.441756 | 0.07 |
| AICAR | 0.82 | BIRB.0796 | 0.73 | Cytarabine | 0.68 | GW843682X | 0.63 |
| AKT.inhibitor.VIII | 0.24 | Bleomycin | 0.75 | Dasatinib | 0.45 | Imatinib | 0.67 |
| AMG.706 | 0.61 | BMS.509744 | 0.67 | DMOG | 0.28 | IPA.3 | 0.75 |
| AP.24534 | 0.83 | BMS.536924 | 0.52 | Docetaxel | 0.28 | JNJ.26854165 | 0.33 |
| AS601245 | 0.38 | BMS.708163 | 0.86 | Doxorubicin | 0.76 | JNK.9L | 0.01 |
| ATRA | 0.81 | BMS.754807 | 0.97 | EHT.1864 | 0.19 | JNK.Inhibitor.VIII | 0.89 |
| AUY922 | 0.71 | Bortezomib | 0.51 | Elesclomol | 0.29 | JW.7.52.1 | 0.69 |
| Axitinib | 0.10 | Bosutinib | 0.83 | Embelin | 0.65 | KIN001.135 | 0.76 |
| AZ628 | 0.66 | Bryostatin.1 | 0.08 | Epothilone.B | 0.32 | KU.55933 | 0.68 |
| AZD.0530 | 0.72 | BX.795 | 0.43 | Erlotinib | 0.13 | Lapatinib | 0.66 |
| AZD.2281 | 0.32 | Camptothecin | 0.59 | Etoposide | 0.69 | Lenalidomide | 0.44 |
| AZD6244 | 0.70 | CCT007093 | 0.74 | FH535 | 0.07 | LFM.A13 | 0.86 |
| AZD6482 | 0.52 | CCT018159 | 0.99 | FTI.277 | 0.98 | Metformin | 0.14 |
| AZD7762 | 0.99 | CEP.701 | 0.80 | GDC.0449 | 0.64 | Methotrexate | 0.44 |
| AZD8055 | 0.53 | CGP.082996 | 0.91 | GDC0941 | 0.46 | MG.132 | 0.37 |
| BAY.61.3606 | 0.79 | CGP.60474 | 0.82 | Gefitinib | 0.55 |  |  |
