# Supplementary material for: Potential prognosis index for m6A-related mRNA in cholangiocarcinoma
Source: BMC Cancer. 2022 Jun 7;22:620. doi: 10.1186/s12885-022-09665-3 (PMC9170563; doi:10.1186/s12885-022-09665-3)
Supplement: Supplementary file 5 — Additional file 5: Supplementary Table S4. Comparison of 22 tumor-infiltrating immune cell types between the low- and high-risk groups. [file 12885_2022_9665_MOESM5_ESM.doc]

Supplementary Table S4. Comparison of 22 tumor-infiltrating immune cell types between the low- and high-risk groups.

| Immune cell type | *t* test | | | Wilcoxan test | | |
| --- | --- | --- | --- | --- | --- | --- |
|  | P value | Adjusted P value | p.format | P value | Adjusted P value | p.format |
| Naïve B cells | 0.52 | 0.52 | 0.52 | 0.77 | 0.77 | 0.77 |
| Memory B cells | 0.17 | 0.17 | 0.17 | 0.53 | 0.53 | 0.53 |
| Plasma cells | 0.88 | 0.88 | 0.88 | 0.88 | 0.88 | 0.89 |
| CD8+ T cells | 0.40 | 0.40 | 0.40 | 0.65 | 0.65 | 0.65 |
| CD4+ memory resting T cells | 0.26 | 0.26 | 0.26 | 0.17 | 0.17 | 0.17 |
| CD4+ memory activated T cells | 0.74 | 0.74 | 0.74 | 0.87 | 0.87 | 0.87 |
| CD4+ naïve T cells | 0.32 | 0.32 | 0.32 | 0.13 | 0.13 | 0.13 |
| Follicular helper T cells | 0.59 | 0.59 | 0.59 | 0.69 | 0.69 | 0.69 |
| Regulatory T cells | 0.02 | 0.02 | 0.02* | 0.02 | 0.02 | 0.02* |
| Gamma delta T cells | 0.51 | 0.51 | 0.51 | 0.37 | 0.37 | 0.37 |
| Resting natural killer (NK) cells | 0.62 | 0.62 | 0.62 | 1.00 | 1.00 | 1.00 |
| Activated NK cells | 0.47 | 0.47 | 0.47 | 0.42 | 0.42 | 0.42 |
| Monocytes | 0.86 | 0.86 | 0.86 | 0.16 | 0.16 | 0.16 |
| M0 macrophages | 0.38 | 0.38 | 0.38 | 0.48 | 0.48 | 0.48 |
| M1 macrophages | 0.82 | 0.82 | 0.82 | 0.99 | 0.99 | 0.99 |
| M2 macrophages | 0.27 | 0.27 | 0.27 | 0.67 | 0.67 | 0.67 |
| Resting dendritic cells | 0.38 | 0.38 | 0.38 | 0.69 | 0.69 | 0.69 |
| Activated dendritic cells | 0.95 | 0.95 | 0.95 | 0.79 | 0.79 | 0.79 |
| Resting mast cells | 0.94 | 0.94 | 0.94 | 0.52 | 0.52 | 0.53 |
| Activated mast cells | 0.47 | 0.47 | 0.47 | 0.92 | 0.92 | 0.92 |
| Eosinophils | 0.27 | 0.27 | 0.27 | 0.16 | 0.16 | 0.16 |
| Neutrophils | 0.16 | 0.16 | 0.16 | 0.53 | 0.53 | 0.53 |

*Significant at P < 0.05.
