# Supplementary figures and images for: Potential prognosis index for m6A-related mRNA in cholangiocarcinoma
Source: BMC Cancer. 2022 Jun 7;22:620. doi: 10.1186/s12885-022-09665-3 (PMC9170563; doi:10.1186/s12885-022-09665-3)

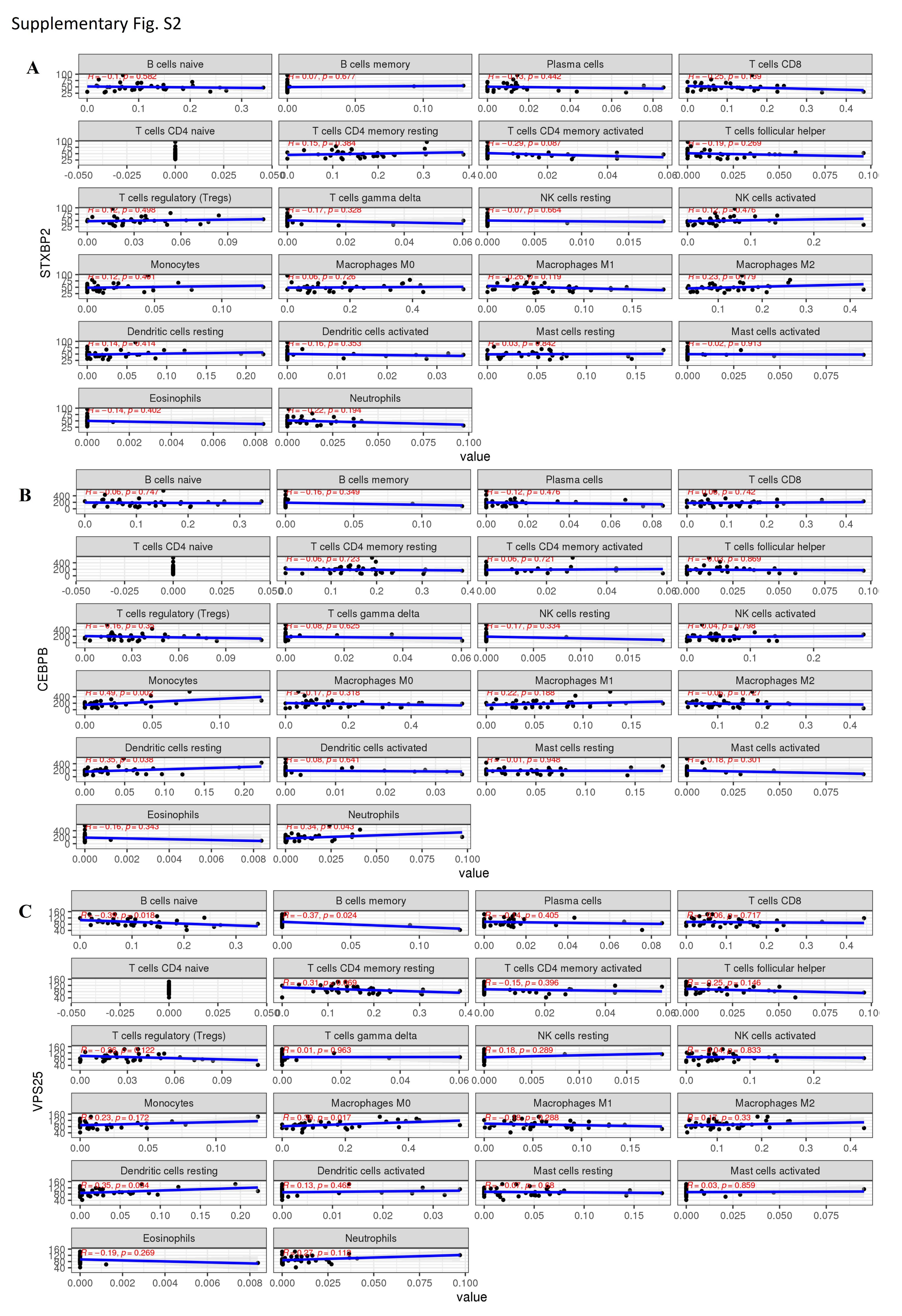

Supplement: Supplementary file 6 — Additional file 6: Supplementary Fig. S2. Correlation between the proportion of tumor-infiltrating cells in the two risk groups with expression of the five m6A-related mRNAs. A) Scatterplot of the relationship between the abundance of the 22 TICs and STXBP2 expression. B) Scatterplot of the relationship between the abundance of the 22 TICs and CEBPB expression. C) Scatterplot of the relationship between the abundance of 22 TICs and VPS25 expression. CEBPB: CCAAT/enhancer binding protein beta; VPS25: vacuolar protein sorting 25 homolog; STXBP2: syntaxin binding protein 2. [file 12885_2022_9665_MOESM6_ESM.jpg]
